# Patterns, Evidence, Gaps and Recommendations Across Studies of Non‐Nursing Tasks: Findings From a Scoping Review

**DOI:** 10.1155/jonm/9885933

**Published:** 2026-10-23

**Authors:** Gaia Magro, Erika Bassi, Stefania Chiappinotto, Gaia Dussi, Alessio Conti, Elisa Ambrosi, Federica Canzan, Giulia Marini, Marco Clari, Alberto Dal Molin, Alvisa Palese

**Affiliations:** ^1^ Department of Medicine University of Udine Udine Italy uniud.it; ^2^ Department of Translational Medicine University of Piemonte Orientale Novara Italy unipmn.it; ^3^ Department of Clinical and Biological Sciences University of Torino Turin Italy unito.it; ^4^ Department of Diagnostics and Public Health University of Verona Verona Italy univr.it; ^5^ Department of Human Sciences University of Verona Verona Italy univr.it; ^6^ Department of Public Health and Pediatrics University of Torino Turin Italy unito.it

**Keywords:** non-nursing activity, non-nursing duties, non-nursing tasks, nonvalue activity, nonvalue task, nurse role, nursing workload, scope of practice, task performance and analysis

## Abstract

**Introduction:**

The increasing demand for healthcare services raises concerns about the sustainability of the nursing workforce. The high prevalence of non‐nursing tasks (NNTs) performed by nurses further exacerbates this issue. However, despite the growing body of research, available evidence has not yet been systematically mapped, limiting healthcare policymakers, leaders and researchers in developing targeted, evidence‐based strategies to mitigate their occurrence and in prioritising research according to the main gaps.

**Aim:**

To map and summarise: (a) concepts, characteristics, antecedents and consequences of NNTs; and (b) gaps in the available evidence and recommendations for future research and policy, as documented in published studies.

**Methods:**

A scoping review was registered in Open Science Framework, conducted using updated Arksey and O’Malley methodology and reported in accordance with PRISMA‐ScR guidelines. Four electronic databases were searched using a predefined search string and eligibility criteria. The Patterns, Advances, Gaps, Evidence for practice and Research recommendations framework was used to organise extracted data, which were analysed using two different methodologies according to the research question: narrative synthesis and thematic analysis. Strategies to ensure rigour were applied.

**Results:**

Twenty‐eight studies published over the last 40 years were retrieved, primarily aimed at measuring the occurrence of NNTs, their consequences and associated factors. Studies were mainly conducted in Europe and the United States and predominantly used cross‐sectional designs based on self‐reported data. Different concepts and definitions of NNTs have been used over time, encompassing administrative and auxiliary work, housekeeping activities and tasks typically performed by other healthcare professionals, which account for approximately 20%–30% of nursing time. Antecedents at the unit level (e.g. staffing shortages) have been most frequently documented, alongside those at the interprofessional level (lack of role clarity and overlapping responsibilities) and at the individual level (issues with role clarity, delegation skills and the desire to provide best possible care for patients). Performing NNTs has negative consequences for patients, nurses and healthcare organisations. The mapped key knowledge gaps and recommendations may inform future research and policies.

**Conclusions:**

The map of knowledge produced to date, together with the identified gaps and documented research recommendations, provides a blueprint for future action. Key priorities include reconceptualising and standardising terminology and measurement approaches in the field, shifting research towards the development and evaluation of strategies to prevent or mitigate NNTs and advancing policies that protect nurses’ time for activities within their professional scope of practice. Addressing these priorities is essential to ensure that nurses’ time is primarily devoted to patient care and to support efficient, sustainable and safe healthcare delivery.

**Implications for Nursing Management:**

These findings inform management by ensuring appropriate staffing and skill mix, clarifying roles and task allocation, redesigning workflows and strengthening the use of support staff and technology.

## 1. Introduction

Given the rising life expectancy and the projected age‐standardised burden of disease between 2022 and 2050 [1], demand for healthcare services is expected to increase, placing additional pressure on already overburdened systems [2]. In this context, the availability of healthcare personnel has become one of the highest priorities in international policy [3]. Nurses, who constitute the largest professional group in healthcare [2], play a central role in meeting these challenges, as they spend most of their working time providing direct and indirect patient care and thereby influence service quality and clinical outcomes [4]. However, the nursing profession is experiencing one of the most severe shortages, which has risen globally from 20.7 million in 2013 (including midwives) to 29.8 million in 2023 [5]. This shortage has been exacerbated by the pandemic, forcing policymakers to take swift and urgent action to increase the supply of nurses worldwide [6].

Within healthcare systems, both nurses and nursing services struggle with significant operational and organisational weaknesses [7]. Key contributing factors include high workloads, inadequate organisational and leadership support [8] and limited opportunities for professional development and recognition [9]. These dynamics place additional strain on nurses’ time and their ability to fulfil their core professional responsibilities, undermining both the quality of care and job satisfaction [10]. Furthermore, the nursing shortage is exacerbated by the amount of time nurses spend on activities outside their scope of practice that are not directly related to patient care or their professional role [11]. To date, various terms have been used to describe these activities, most commonly non‐nursing tasks (NNTs) [12]. Conceptually, these tasks have been described as typically unrelated to the knowledge and qualifications gained during nurses’ formal education and as diverting nursing time from their expected core competencies [13]. Thus, in addition to exacerbating the nursing shortage, NNTs contribute to increased unfinished nursing care, poorer perceptions of the work environment, greater job dissatisfaction and detrimental effects on professional recognition, role definition and overall reputation [13]. Consequently, the occurrence of NNTs may reduce the attractiveness of the nursing profession to prospective entrants and increase nurses’ intention to leave the profession, thereby further exacerbating the nursing shortage [13].

Preventing and mitigating the occurrence and burden of NNTs is a priority for policymakers, professional bodies, healthcare organisations and regional and local nursing services. However, despite the growing body of research produced to date, the available evidence has not yet been synthesised in a comprehensive review describing the key characteristics, temporal trends, antecedents and consequences of NNTs. This evidence gap limits the ability of healthcare leaders and policymakers to develop and implement targeted, evidence‐based strategies to prevent/mitigate NNTs, value the nursing workforce and ensure that nurses can focus on activities within their professional scope of practice. This lack also affects the establishment of timely research priorities in the field.

### 1.1. Aims

The aim was to map and summarise: (a) the concepts, characteristics, antecedents and consequences of NNTs and (b) the gaps in the available evidence and the recommendations for future research and policy, as reported in published studies.

## 2. Methods

### 2.1. Study Design

A scoping review was conducted following the six‐step methodology of Arksey and O’Malley [14], as updated [15, 16]. The research protocol was developed by the Ricerca Infermieristica Clinica *e* per l’Innovazione organizzativa *e* formativa (RICI, Clinical Nursing Research and Organisational and Educational Innovation) consortium (Supporting Table 1), registered in the Open Science Framework, publicly available at 10.17605/OSF.IO/JUSCR, and published [17]. The Preferred Reporting Items for Systematic Reviews and Meta‐Analyses extension for Scoping Reviews (PRISMA‐ScR) [18] was then followed to report the results (Supporting Table 2), and the Patterns, Advances, Gaps, Evidence for practice and Research recommendations (PAGER) framework [19] was used for data charting and synthesis.

### 2.2. Step 1: Research Questions

Two main review questions were addressed:

1. ‘What are the main patterns of the studies on NNTs that have been published to date?’
2. ‘What advances, gaps and recommendations have been documented to date regarding NNTs, their antecedents and their consequences?’

According to the PCC framework [20],

- the Population comprised nurses;
- the Concept included NNTs and all related concepts and definitions identified by two researchers (GMag and SC) through a preliminary, nonsystematic literature search (Table 1). During a subsequent research team meeting, these concepts were critically examined, particularly with regard to their conceptual boundaries and potential areas of overlap. While acknowledging that these concepts may partially overlap, the researchers recognised that they are not synonymous and represent distinct dimensions of nursing work. Based on this analysis, the search strategy was designed to incorporate the identified terms;
- the Context encompassed any setting in which nurses work (e.g. hospitals, community settings and intermediate care).

**TABLE 1:** Non‐nursing tasks: concepts and definitions available in literature.

Concept(s)	Definitions, reference(s)
Non‐care‐related activities	Not related to patient care but significantly affecting the nursing workload [21]
Non‐nursing clerical functions	All activities not requiring nursing expertise [22]
Non‐nursing activities	Tasks beyond traditional RN roles, including managing inventories, scheduling and conducting evaluations [23]
Non‐nursing duties	Tasks that are not related to direct patient care, or belong to other positions, but which relieve the nurse, so that she/he can devote herself/himself to direct patient care [24]
Non‐nursing functions	Non‐clinical activities often performed by RNs that should or could be performed by support service staff or nurse aides [25]
Non‐nursing tasks	Tasks that do not require professional nursing training and should be assigned to other staff; they are considered an obstacle to the provision of care management because they are performed by professional nurses outside their scope of practise [26]
Non‐professional tasks	Activities not requiring professional skills and not related to direct patient care [27]
Non‐RN care	The tasks performed at the bedside or away from bedside that could be performed by emergency department personnel other than a licenced nurse or licenced physician [28]
Non‐value‐added activity	Include the activities that do not affect care, such as waiting or searching for equipment [29]
Non‐value‐added tasks	Activities that do not directly benefit the patient and are not necessary for care delivery [30]
Non‐value‐added time	Time spent on tasks that do not directly contribute to patient care or outcomes, including delays, rework, unnecessary searches and inefficient processes [31]

Abbreviation: RN, registered nurse.

### 2.3. Step 2: Search Strategy

An initial search of the MEDLINE database (via PubMed) was conducted in November 2024 (GMag) to identify subject headings and keywords and refine the search strategy. Subsequently, MEDLINE (via PubMed), CINAHL (the Cumulative Index to Nursing and Allied Health Literature) and the Cochrane Database of Systematic Reviews and Scopus were searched up to 11 November 2024. No restrictions were placed on publication date to ensure a comprehensive map of the literature. The following terms were used and combined: “nurs∗,” “nursing staff,” “non‐nurs∗ task,” “non nurs∗ task,” “non‐nurs∗,” “non nurs∗,” “nurs∗ activit∗,” “non‐nurs∗ activit∗,” “non‐value task” and “non‐value activit.∗ Context was not considered in the search strategy, to capture all settings.

All terms and free‐text words were integrated into the search string using the Boolean operators AND and OR. Search functions [e.g. truncation (∗), phrase searching with quotation marks (“ ”), use of parentheses and MeSH terms] were used to broaden or narrow the search. The full search strategies applied in the databases and the records identified are reported in Table 2.

**TABLE 2:** Search strategies applied in databases and records identified (11 November 2024).

Database(s)		No. of records
PubMed		
Population	“Nurses”[Mesh] OR “Nursing Staff”[Mesh] OR nurs∗	1,210,108
Concept	“non‐nurs∗ task” OR “non nurs∗ task” OR “non‐nurs∗” OR “non nurs∗” OR “nurs∗ activit∗” OR “non‐nurs∗ activit∗”	2760
Population AND Concept	(“Nurses”[Mesh] OR “Nursing Staff”[Mesh] OR nurs∗) AND (“non‐nurs∗ task” OR “non nurs∗ task” OR “non‐nurs∗” OR “non nurs∗” OR “nurs∗ activit∗” OR “non‐nurs∗ activit∗”)	2700
CINAHL EBSCO		
Population	“nurs∗” OR “nursing staff”	1,005,243
Concept	“non‐nurs∗ task” OR “non nurs∗ task” OR “non‐nurs∗” OR “non nurs∗” OR “nurs∗ activit∗” OR “non‐nurs∗ activit∗” OR “non‐value task” OR “non‐value activit∗”	2329
Population AND Concept	(“nurs∗” OR “nursing staff”) AND (“non‐nurs∗ task” OR “non nurs∗ task” OR “non‐nurs∗” OR “non nurs∗” OR “nurs∗ activit∗” OR “non‐nurs∗ activit∗” OR “non‐value task” OR “non‐value activit∗”)	2299
SCOPUS		
Population	“nurs∗” OR “nursing staff”	3,275,228
Concept	“non‐nurs∗ task” OR “non nurs∗ task” OR “non‐nurs∗” OR “non nurs∗” OR “nurs∗ activit∗” OR “non‐nurs∗ activit∗” OR “non‐value task” OR “non‐value activit∗”	3055
Population AND Concept	(“nurs∗” OR “nursing staff”) AND (“non‐nurs∗ task” OR “non nurs∗ task” OR “non‐nurs∗” OR “non nurs∗” OR “nurs∗ activit∗” OR “non‐nurs∗ activit∗” OR “non‐value task” OR “non‐value activit∗”)	1462
Cochrane		
Population	“nurs∗” OR nursing NEXT staffing OR Nurses OR Nursing	81,978
Concept	“non‐nurs∗” NEXT task∗ OR non NEXT nurs∗ NEXT task∗ OR “non‐nurs∗” OR non NEXT nurs∗ OR nurs∗ NEXT activit∗ OR “non‐nurs∗” NEXT activit∗ OR “non‐value” NEXT task∗ OR “non‐value” NEXT activit∗	192
Population AND Concept	(“nurs∗” OR nursing NEXT staffing OR Nurses OR Nursing) AND (“non‐nurs∗” NEXT task∗ OR non NEXT nurs∗ NEXT task∗ OR “non‐nurs∗” OR non NEXT nurs∗ OR nurs∗ NEXT activit∗ OR “non‐nurs∗” NEXT activit∗ OR “non‐value” NEXT task∗ OR “non‐value” NEXT activit∗)	192

*Note:* CINAHL EBSCO, Cumulative Index to Nursing and Allied Health Literature; N, number.

### 2.4. Step 3: Study Selection

Studies were included if they were (1) primary quantitative or qualitative studies, mixed‐methods studies or secondary studies in the form of reviews or evidence‐based guidelines published in peer‐reviewed journals; (2) focused on NNTs or related concepts; and (3) written in Italian or English. Exclusion criteria encompassed editorials, commentaries, letters, conference abstracts, books, dissertations, studies not focusing on NNTs or related concepts and studies concerning other populations (e.g. nursing students). The Rayyan software [32] was used during the screening phase of titles and abstracts to organise the sources and increased efficiency.

Initially, duplicate articles were removed manually. The first researcher (GMag) screened titles and abstracts against the inclusion criteria. The screening process was independently double‐checked by researchers (EB, SC, GD, AC, MC and GMar), and any discrepancies were resolved through discussion until consensus was reached. In the event of disagreement, an additional researcher (AP) was consulted.

Sixty studies were identified as eligible, and their reference lists were examined to identify additional sources; a total of five articles were not available in full text (Supporting Table 3). The full texts retrieved were then assessed in accordance with the inclusion criteria by GMag and independently screened by two other researchers (GD and SC). At this stage, disagreements were again resolved through consensus under the supervision of another researcher (AP). A comprehensive flow diagram [33] was completed, resulting in 28 included studies (Figure 1, Supporting Table 3). In line with scoping review methodology [14–16], critical appraisal of the quality of the included studies was not performed.

**FIGURE 1:**
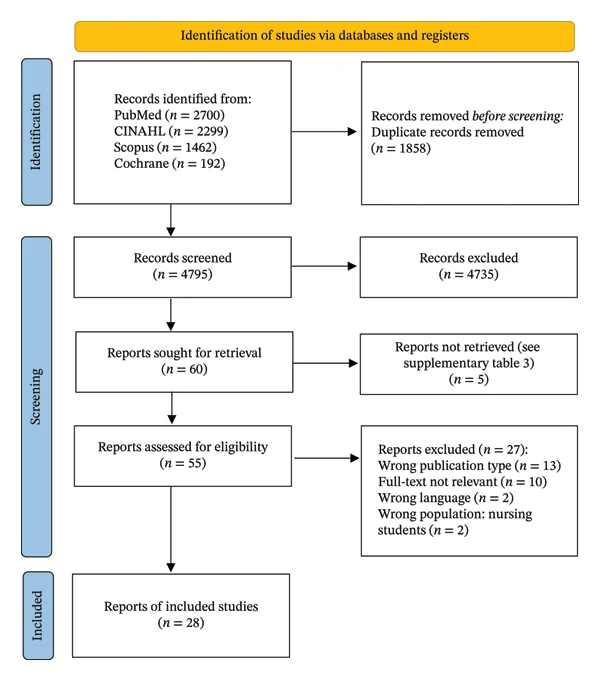
PRISMA 2020 flow diagram for new systematic reviews which included searches of databases and registers [33].

### 2.5. Step 4: Data Extraction

Based on the recommendations of the Joanna Briggs Institute [20], a grid was developed to extract information relevant to the review questions. The grid was piloted in two studies, and no changes were required. To answer:

1. the first question, namely, ‘What are the main patterns of the studies on NNTs that have been published to date?’, data were extracted regarding (a) author(s) and affiliation, year of publication and the country in which the study was conducted (according to the affiliation of the author(s), if not explicitly stated); (b) aim(s) of the study; (c) design; and (d) main characteristics of the study, such as setting, participants and data collection instruments and processes;
2. the second question, namely, ‘What advances, gaps and recommendations have been documented to date regarding NNTs, their antecedents and consequences?’, data were extracted regarding (a) ‘Advances’, such as concepts of NNTs used in the studies, documented NNTs frequency and type, and antecedents and consequences and (b) ‘Gaps’ in the available evidence and ‘Recommendations’ for policy and research, as documented in the studies.

Data extraction was performed by GMag and then reviewed by two other researchers (SC and GD) and audited by a fourth (AP).

### 2.6. Step 5: Data Synthesis and Reporting

The extracted data were summarised narratively [34], according to the review questions and following the PAGER framework [19]. First, the ‘Patterns’ identified in the studies were summarised (years of publication, countries, study designs, aims, participants, tools and data collection processes). Second, evidence and advances produced to date, in terms of the conceptual definitions of NNTs or related terms and the main features of NNTs in relation to type and frequency, were described narratively. Antecedents and extracts were categorised through thematic analysis [20], as methodologically recommended for scoping reviews [16, 35]. The data extracted from the included studies were read and independently grouped by three researchers into three organisational levels for antecedents: unit, interprofessional and individual. A senior researcher resolved any disagreements. A similar process was applied to data extracted regarding NNTs outcomes, which were categorised through thematic analysis [20] as consequences at the patient, nurse and organisation levels. Finally, gaps and recommendations for research and policy were summarised narratively.

### 2.7. Step 6: Consultation of Stakeholders

Members of the RICI consortium (Supporting Table 1) were purposively selected [36] to ensure representation of key stakeholder groups, including a clinical nurse, a nurse manager and a nurse researcher/educator. They took part in a consultation meeting to discuss the review findings, assess their relevance across different contexts (nursing education, research and practice) and provide additional insights to support their interpretation. The feedback collected informed the discussion and the interpretation of the data summary that emerged from the review.

### 2.8. Rigour

Several strategies were adopted to ensure methodological rigour. The scoping review protocol was conceptualised, methodologically developed and subsequently refined through iterative discussions with the wider research team. The protocol was also prospectively registered, is publicly available and has been published [17], ensuring that the review process was transparent and verifiable. A comprehensive search strategy incorporating multiple terms and keywords was developed to capture the conceptual and terminological complexity of the field (Table 1), including the partial overlap among related concepts. The strategy was applied across multiple databases to maximise the identification of relevant studies and minimise publication bias [37]. Study selection was conducted independently by researchers at each stage, followed by systematic cross‐checking to ensure completeness, accuracy and consistency in identifying eligible studies, while reducing the risk of selection bias. Data extraction, analysis, narrative synthesis and thematic analysis were performed independently by three researchers (see authors) to ensure accuracy and consistency. The senior investigator supervised the process and was consulted whenever disagreements arose. Regular team meetings were held throughout the data extraction phase to ensure coordination and methodological consistency while data curation and validation involved the wider research team. Overall, data analysis was conducted in accordance with the PAGER framework [19], and the methodological conduct and reporting of the scoping review followed the PRISMA‐ScR guidelines [18, 33].

## 3. Results

### 3.1. Research Patterns

A total of 28 studies published between 1984 [22] and 2024 [26, 38] were included (Supporting Table 4), and their main patterns are summarised in Table 3.

**TABLE 3:** Research patterns on NNTs: summary of the included studies (*N* = 28).

Pattern(s)	Category	No. of studies, references
Years of publication	< 2000	9 [22, 24, 25, 39–44]
	2000–2004	1 [45]
	2005–2009	4 [28, 30, 31, 46]
	2010–2014	1 [47]
	2015–2019	8 [21, 23, 29, 48–52]
	2020–2024	5 [26, 27, 38, 53, 54]

Country	United States	7 [22, 25, 28, 30, 31, 43, 50]
	United Kingdom	6 [24, 39–42, 45]
	Canada	3 [29, 44, 51]
	Italy	3 [40–42]
	Jordan	2 [26, 38]
	China	1 [27]
	Egypt	1 [54]
	Finland	1 [47]
	Greece	1 [46]
	Iran	1 [21]
	South Africa	1 [53]
	New Zealand	1 [23]

Study design	Cross sectional	11 [21, 22, 29, 31, 42, 45, 47, 49, 52–54]
	Observational	5 [28, 30, 39, 41, 46]
	Qualitative	3 [26, 38, 48]
	Review	2 [23, 40]
	Quasi‐experimental	1 [25]
	Time‐motion	1 [50]
	Panel analysis	1 [27]
	Qualitative improvement	1 [51]
	NR	3 [24, 43, 44]

Setting of the study	Mixed care units	10 [22, 24, 26, 30, 31, 38, 43, 47, 51, 54]
	Medical‐surgical departments	4 [25, 27, 50, 52]
	Intensive care units	3 [21, 42, 45]
	Other (e.g. post anaesthesia)	3 [28, 41, 46]
	Long‐term care units	2 [23, 29]
	Paediatric wards	2 [39, 44]
	NR	4 [40, 48, 49, 53]

Participants	Nurses	28, [all references]
	Licensed practical nurses	3 [22, 29, 51]
	Nursing assistants	1 [31]
	Health care support worker	1 [39]
	Patients care attendants	1 [51]
	Resident aides	1 [29]
	Other (e.g. support staff)	1 [31]

Data collection	Self‐report questionnaire	12 [22, 24, 25, 27, 42–45, 47, 49, 51, 53]
	Observational methods	8 [21, 28–30, 39, 41, 46, 50]
	Validated tools (RN4CAST questionnaire, MISSCARE survey)	2 [52, 54]
	Focus group	2 [26, 38]
	Semistructured interviews	2 [39, 48]

*Note:* MISSCARE, missed nursing care survey; N, number; NNTs, Non‐nursing tasks; RN4CAST, Registered nurse forecasting.

Abbreviation: NR, not reported.

The studies addressed three main aims: (a) quantifying the amount and type of NNTs performed by nurses in different clinical settings (*N* = 16) [21, 22, 27, 29, 39, 41–43, 45, 46, 49–54]; (b) investigating nurses’ experiences, perceptions and the professional and clinical consequences associated with performing NNTs (*N* = 9) [23, 25, 26, 28, 38, 40, 44, 47, 48]; and (c) examining organisational, systemic and managerial factors contributing to NNTs and identifying interventions to mitigate their effects (*N* = 3) [24, 30, 31].

They were mainly conducted in Europe (*N* = 11) [24, 39–42, 45–49, 53], followed by the United States (*N* = 7) [22, 25, 28, 30, 31, 43, 50], Asia (*N* = 4) [21, 26, 27, 38], Canada (*N* = 3) [29, 44, 51], Africa (*N* = 2) [52, 54] and New Zealand (*N* = 1) [23] (Table 3). Cross‐sectional (*N* = 11) [22, 24, 26, 28, 30, 31, 38, 43, 47, 51, 54] and observational designs (*N* = 5) [28, 30, 39, 41, 46] were prevalent. The studies were conducted in mixed settings (*N* = 10) [22, 24, 26, 30, 31, 38, 43, 47, 51, 54], involving nurses, while five studies [22, 29, 31, 39, 51] also included other healthcare professionals. The number of participants ranged from 3 [39] to 1498 [27], with some studies (*N* = 10) reporting no data [22, 24, 28, 29, 31, 42, 43, 45, 46, 51].

Data were collected mainly using self‐report questionnaires (*N* = 12) [22, 24, 25, 27, 42–45, 47, 49, 51, 53]; only two studies based their data collection on validated instruments, namely, the RN4CAST questionnaire and the MISSCARE survey [52, 54]. Different observational methods were used in eight studies [21, 28–30, 39, 41, 46, 50]. Qualitative studies used focus groups [26, 38] and semistructured interviews [39, 48].

### 3.2. Advances

#### 3.2.1. Concepts and Definitions

NNTs have been described using varied concepts across studies (Table 4). The earliest term identified was ‘non‐nursing clerical functions’ [22], while early studies predominantly used ‘non‐nursing duties’ [24, 39, 40, 42] and ‘non‐value‐added tasks/time/activities’ [29–31, 53]. The term ‘NNTs’ became more common from 2015 onwards [26, 38, 48, 49, 52–54]. Their definitions were similarly heterogeneous (Supporting Table 4). NNTs were defined as activities performed outside or below the traditional nursing scope of practice [26, 38, 48, 49, 52, 53] or as tasks not requiring professional nursing skills [54]. Other studies described ‘non‐nursing duties’ as activities that do not involve direct patient care or do not require nursing qualifications [24, 39, 40, 42, 46, 51], whereas ‘non‐value‐added activities/time/tasks’ were defined as activities that do not benefit patients or contribute to care [29–31, 53]. Additional terms included ‘non‐care‐related activities’, referring to activities unrelated to patient care [21]; ‘non‐nursing functions’, referring to tasks that should be performed by support staff [25]; ‘non‐RN care’, referring to tasks performed away from the bedside [28]; ‘non‐professional tasks’, referring to activities that do not require professional skills or direct patient care [27]; ‘non‐nursing activities’, referring to tasks beyond the traditional nursing role [23]; and ‘non‐nursing clerical functions’, referring to clerical tasks that do not require nursing expertise [22]. The remaining studies did not provide a conceptual definition [41, 43–45, 47, 50].

**TABLE 4:** Advances in the evidence available on non‐nursing tasks: map of the studies published to date (*N* = 28).

Studies	Concept	Types, measure(s), and time burden of NNTs			Antecedents: levels			Consequences: Levels		
		Type	Measure	Time burden	Unit	Interprofessional	Individual	Nurses	Patient care	Organisation
Ahmadishad et al. (2019) [21]	Non‐care‐related activities	/	%	110.9 min (30.8%) of morning shift	Type of unit Nurse–patient ratio Weakness of managerial supervision Organizational policy		Level of professional commitment and behavioural patterns Limited familiarity with job description	/	/	/

Al‐Akash et al. (2024) [38]	Non‐nursing tasks (NNTs)	Clerk and management, transporting, physician	/	/	Staffing shortages High workload Role of management in task allocation Transparency in task distribution	Unclear communication channels Confusion on nurses’ duties and responsibilities	Lack in defining clear nurses’ role	Reduction of nurses’ effectiveness and of professional growth Hindering ability to fully grasp clinical difficulties and make decision Internal conflicts and demotivation Intention to leave	/	Reduction of productivity Quality of healthcare services

Aldarawsheh et al. (2024) [26]	NNTs	Management and clerical, transporting, physician, communication and coordination, tasks performed by other healthcare professionals	/	/	Staffing shortages Limited resources Weakness of nursing managements (leading to assignment of NNTs to nurses)	/	Weakness of education Performed NNTs as a favour of other colleagues	Physical and mental health strain Reduction of professional development Internal struggles and disheartenment	Deterioration of nursing care Challenges to prioritize or provide patient care Impaired nursing care Quality of patient care	Reduction of efficiency, productivity

Bekker et al. (2015) [52]	NNTs	Delivering/retrieving food trays, housekeeping, transporting patients, ordering supplies, obtaining equipment, discharge referral and transport arrangements	Mean of activities	/	High workload High unit occupancy Nurse to patient ratio Unfavourable practice environment Poorly functioning support services	/	/	Dissatisfied with job	Leave some nursing tasks left undone	/

Fulbrook (1999) [39]	Non‐nursing duties	Housekeeping, equipment preparation, measuring, administrative tasks	%	60% of staff time	Understaffing of support roles	Lack of formal role delineation	/	Underutilisation of qualified expertise	Potential compromise of quality of care Low capacity for direct patient care	Missed opportunities for nursing‐led service development

Grosso, Tonet, Bernard, Corso, et al. (2019) [48]	NNTs	administrative tasks, activities required less/higher education	/	/	Cost containment Staffing shortages Skill mix revisions Unit routines Culture Implicitly force to perform some function	Lack of communication and discussion	Desire to meet patients’ need Ensure comfort and well‐being of patients Changing from vocational to academic education	Confusion and ambiguity Frustration Erosion of nursing competences Disaffection Intention to leave Accumulation of extra hours Feeling tension towards healthcare with less flexibility	Missing planning nursing care Increased need of care	/

Grosso, Tonet, Bernard, De Marchi, et al. (2019) [49]	NNTs	/	%	32.6% of shift time	Lack of support and auxiliary staff Changes in staff mix Organizational structure Culture Absence of non‐nursing personnel	/	/	Reduction of job satisfaction Augmented the perception of time waste Risk of burnout and team conflict	Augmented missed care	/

Grosso et al. (2021) [53]	NNTs Non‐value added (NVA) activities	Administrative, auxiliary, allied healthcare professional tasks	%	32.6% of total shift work	Working in hospitals Functional model Inadequacy of nursing resources High workload Pressures from organizational culture	/	Perceived need to compensate	Reduction of job satisfaction Higher intention to leave unit Professional burnout Perception of wasted professional time	Missed nursing care (MNC)	/

Hammad et al. (2021) [54]	NNTs	Delivering food trays, arranging discharge referrals, transporting patients, cleaning patients’ room, obtaining supplies, clerical tasks	/	/	/	/	/	/	MNC	/

Hamm‐Vida (1990) [43]	/	Housekeeping, transporting, pharmacy, secretarial	%	67.5 h (night shift) 35.82 h (afternoon shift) 3.84 h (morning shift)	/	/	/	/	/	/

Harrison & Nixon (2002) [45]	/	Cleaning and preparing bed spaces, ordering/putting away stores, filling out requisition slips, checking general equipment, answering phones	%	4% of shift time ↑ 6% late shift ↓ 3% morning/night shift	Inadequate support staff	/	/	Inefficiency Higher workload for nurses	Low focus on direct patient care	/

Hayes (1994) [25]	Non‐nursing functions	Clerical/receptionist, housekeeping, making beds, dietary, pharmacy	/	/	Time	Lack in the role of nurse aides	Shorter length of employment Younger Register Nurses (RNs) Inexperience	Overall job satisfaction Frustration	/	/

Hinno et al. (2011) [47]	/	Delivering/removing food, transporting patients, making beds, changing bed linen, administrative (appointments, ordering bedlinen and laboratory test, making rosters, organising patients)	/	/	High patient‐to‐nurse ratio Inadequate support staff Local context	/	/	Reduction of time for crucial nursing activities	/	/

Hobgood et al. (2005) [28]	Non‐RN care	Ordering, transporting patients, preparing patients meal	%	6.8% of time	Patient per nurse Emergency Department (ED) acuity index High workload	/	/	/	/	/

Johnson (1989) [40]	Non‐nursing duties	Housekeeping, transportation, dietary, restocking, cleaning, reordering	/	/	High patient turnover	/	/	Reduction of job satisfaction	/	/

Johnson (1991) [41]	—	Restocking, cleaning, answering phones, carrying nonurgent equipment, and routine preparation of operating room	%	11% of time ↑ NNT in evening/ weekend shift	Lack of ancillary staff (evening/weekend shifts) Staffing shortages	/	/	/	/	/

Kearney et al. (2016) [51]	Non‐nursing duties	Answering phones, housekeeping, food services, transportating patients	/	/	Limited availability of support staff	/	/	Lower job satisfaction	Low time for direct patient care Augmented potential medical errors	/

Kiekkas et al. (2005) [46]	Non‐nursing duties	Cleaning spaces, transferring patients/equipment	%	2.3% of time (8.6% in clerical, 9.3% in documentation)	Inadequate nurse–patient ratio Staffing shortages	/	/	/	Low time for patient assessment Low direct care Low psychological support, that impacting care quality	/

Liu et al. (2021) [27]	Non‐professional tasks	Discharge, referral, coordination, obtaining equipment, cleaning, transporting patients, delivering food	% of activities	/	/	/	/	/	/	/

MacLeod (1985) [24]	Non‐nursing duties	Washing patients’ clothing, carrying from stores, cleaning, unpacking laundry, transporting patients, sorting/filling notes	/	/	Nursing shortage Workloads Patient turnover Lack of ancillary support High workload pace	/	Level of education to performing non‐nursing duties	/	Reduction of patient care and quality of care	Increasing of cost of healthcare professionals

McCloskey (2015) [29]	NVA activities	Look for charting, restocking, walking, getting equipment	%	13.9% (mean) of shift time	/	/	/	/	/	/

Montayre & Montayre (2017) [23]	Non‐nursing activities	/	/	/	Staffing shortages	Inadequate delegation	Poor role clarity	Overlapping roles Erosion of nurses’ expertise	Low focus on direct care	/

Norrie (1997) [42]	Non‐nursing duties	Cleaning/re‐provisioning, replacement bed‐unit	%	7% of time	High turnover of patients Lack of support workers (morning shift)	/	/	/	/	/

Sabo (1990) [44]	/	Answering phones, completing requisitions, taking messages, delivering/collecting trays	/	/	Nursing shortage	/	/	Dissatisfaction Risk of burnout High turnover	No responses to patients’ needs	/

Storfjell et al. (2009) [31]	NVA time	Coordinating care, managing clinical records, documentation and equipment search	%	38% of shift time (∼$660 k–$660‐756 k/year per unit	Fragmented workflows Frequent handoffs Poor access to equipment, supplies, or medications Difficult process of admission/transfer/discharge Inefficient electronic health records (multiple systems, redundancy) Fluctuating staffing Time‐consuming shift reports	Inadequate interdisciplinary communication	/	Staff dissatisfaction Risk of burnout	Low time for direct patient care High risk of adverse events High patient safety Disconnection between staffing and care needs	High operational costs Documentation overload

Upenieks et al. (2007) [30]	NVA tasks	/	%	16.7–21% of time	Inadequate inclusion of support staff in staffing calculations Physical layout of units (e.g., distance to nurses’ station) Lack of standardization in patient classification systems Focus on ratios without accounting for task complexity or indirect care	/	/	Overburdened nurses with indirect/non‐nursing duties Risk of burnout	Low time for direct patient care	Inefficiency due to high administrative/documentation burden Inconsistency in workload distribution

Yauger (1984) [22]	Non‐nursing clerical functions	Telephone duties, transcribing order, reception duties	%	1.4‐12% of shift time 657.4 h	Nursing shift (late afternoon and early evening)	/	/	/	Compromising patient care activities, both direct and indirect	/

Yen et al. (2018) [50]	/	Transferring record, copying documents, food trays, housekeeping, phone call	%	10% of shift time	Insufficient staffing Inattention by other departments	Inattention by other staff	/	Dissatisfaction Increasing nurses’ stress	Low time for patient care	Augmented work inefficiency

*Note:* PAGER, Patterns, Advances, Gaps, Evidence for practice, Research recommendations.

Abbreviations: ED, emergency department; MNC, missed nursing care; NNT, non‐nursing task; NVA, nonvalue added; RN, registered nurse.

#### 3.2.2. Types, Time and Temporal Burden of NNTs

Overall, 24 studies described types of NNTs [21, 22, 24–29, 38–47, 49–54] while other studies (*N* = 15) documented the time (measured or estimated) spent on these activities and/or the proportion of nurses’ shifts devoted to them [21, 22, 28–31, 39, 41–43, 45, 46, 49, 50, 53]. Three studies reported measures of their organisational costs [24, 31, 40] (Table 4 and Supporting Table 4).

Activities classified as NNTs mainly comprised:

a. tasks related to the unit, such as administrative, auxiliary or housekeeping duties [22, 25, 26, 38–41, 44–49, 51–54]. The most common activities considered NNTs were answering the phone [22, 41, 44, 49, 52], filling in charts [25, 40, 41, 44, 51], ordering supplies [40, 41, 47, 52] and auxiliary activities, such as transporting patients [27, 38, 46, 47, 51, 52, 54], delivering food trays [40, 44, 46, 47, 49–52, 54] and housekeeping [25, 39–42, 44, 46, 47, 51, 52, 54];
b. tasks related to other healthcare professions, namely, activities expected of physiotherapists, respiratory therapists, dietitians and physicians but performed by nurses [26, 38, 48, 53].

Six studies focused exclusively on activities classified as NNTs [22, 39, 43, 48, 52, 53], and seven examined these activities in comparison with other activities such as direct care and personal activities [21, 27–30, 42, 45].

In the measurements, NNTs were expressed as a percentage of the NNTs performed by nurses (*N* = 15) [21, 22, 28–31, 39, 41–43, 45, 46, 49, 50, 53] with reference to: (a) the work shift [21, 31, 43, 45, 49, 50, 53]; (b) working hours [22, 29, 30, 39]; or (c) not specified [28, 41, 42, 46]. Overall, the proportion of working time devoted to NNTs ranged from 1.4% to 60% [21, 22, 28–31, 39, 41–43, 45, 46, 49, 50, 53]. Studies published in the last 10 years reported that these activities accounted for 10%–32.6% of nurses’ working time [21, 29, 49, 50, 53]. Two studies described the percentages or means of NNTs performed by nurses [27, 52] without reporting specific data.

Only three studies reported NNT‐related organisational costs, either as cost estimates (e.g. loss of £130,000 per year, the amount of the nursing budget spent on paying nurses to perform auxiliary work [24, 40]) or as actual costs (e.g. costs of non‐value‐added activities: over $1 million per year in medical‐surgical units [31]).

#### 3.2.3. Antecedents

Twenty‐four studies identified antecedents associated with NNTs (Table 4) at unit, interprofessional and individual levels.

Unit‐level antecedents were most frequently reported (*N* = 24) and included staffing shortages, high workload, inadequate nurse‐to‐patient ratios, limited availability of support staff, changes in skill mix, organisational routines and policies and unclear job descriptions [21–24, 26, 28, 30, 38, 39, 41, 42, 44–53]. In addition, weak management oversight, lack of time and poor governance in task allocation were reported [21, 25, 26, 38]. Some studies identified patient complexity and turnover, and admission and discharge processes, as contributing to the occurrence of NNTs [28, 30, 31, 40, 42, 47, 53]. One study identified an inefficient electronic system that was not integrated with clinical documentation as an antecedent; this required nurses to report the same data in more than one place and to search for information in different locations [31].

Interprofessional‐level factors were documented by five studies [23, 25, 31, 38, 48], which reported a lack of role clarity and overlapping responsibilities among healthcare professionals [23, 25, 38]. Inefficient communication, whether due to informal, inadequate or hierarchical channels, combined with the absence of delegation frameworks and collaborative decision‐making mechanisms to maintain continuity of care, resulted in fragmented coordination and unclear accountability for task allocation [23, 31, 38, 48].

Individual‐level antecedents were reported in eight studies and included nurses’ lack of experience, the lack of a clearly defined nursing role and the lack of familiarity with the role [21, 25, 38]; issues in the delegation of competences [23]; and the desire to carry out activities in favour of patients and promote their well‐being [48] or to compensate for structural deficiencies [53]. Nurses’ education [24, 26, 48] also influenced the propensity of nursing staff to perform NNTs.

#### 3.2.4. Consequences

Twenty‐two studies reported outcomes associated with NNTs at nurse, patient care and organisational levels (Table 4).

Both nurse‐related and patient care outcomes were reported in 17 studies: NNTs have been shown to reduce job satisfaction and professional development; increase emotional exhaustion, stress, burnout and role confusion; and lead to erosion of competencies and underutilisation of nurses’ expertise [23, 25, 26, 30, 31, 38–40, 44, 45, 47–53]. Moreover, NNTs have been associated with perceptions of wasted professional time, working additional hours to perform nursing activities and having less time available for direct patient care [30, 45, 48, 49, 53]. Conflicts and tensions within the team have also been reported [38, 48, 49], as well as staff turnover and intention to leave [38, 44, 48, 53].

Consequences for patient care outcomes mainly included challenges in nursing care planning, reduced time spent on direct patient care activities and difficulties in responding to patients’ needs [22, 23, 26, 30, 31, 39, 44–46, 48, 50, 51]. Other outcomes were related to deficits in the quality of patient care [24, 26, 39, 46], including missed nursing care or tasks left undone [49, 52–54] and an increased risk of adverse events and medical errors [31, 51].

Organisational outcomes were less frequently reported (*N* = 7) and included reduced productivity and efficiency, inconsistent workload distribution, increased operational or staffing costs, documentation burden and missed opportunities for the development of nursing services [24, 26, 30, 31, 38, 39, 50].

### 3.3. Gaps, Research and Policy Recommendations

#### 3.3.1. Gaps

As summarised in Table 5, several gaps have been identified in this field of study: a lack of multicentre, large‐scale research [21, 53]; issues with standardised definitions and conceptualisation of NNTS [21, 53]; limited data on temporal differences in NNTs across shifts, such as night shifts [21, 22, 41, 43, 45, 53]; and a scarcity of interventional studies [27, 30]. Gaps in organisational and policy analysis, including policies on task allocation and support staff [23, 26, 38, 48], have also been documented. Moreover, gaps in the available knowledge concern the effects of NNTs on patient outcomes and adverse events [23, 30, 47], the psychological well‐being of nurses [26, 30, 31, 44, 48, 49, 53], the occurrence of missed care [54] and costs [24, 31, 40].

**TABLE 5:** Gaps, Research and Policy Recommendations as documented in the studies included (*N* = 28).

Gaps, [reference(s)]	Research recommendations, [reference(s)]	Policy recommendations, [reference(s)]
Lack of multicentre and large‐scale studies assessing the time distribution of nurses’ activities [21, 53] Limited analysis of temporal variations in NNTs during nursing shifts [21, 22, 41, 43, 45, 53] Limited intervention‐focused studies [27, 30] Lack of standardised definitions and categorisation of task types [21, 53] Limited exploration of NNTs as a process influenced by interprofessional boundaries [53] Lack of organisational and policy‐level analysis [38] Absence of policies regulating task allocation and support staffing levels [23, 26, 38, 48] Lack of investigations regarding the relationship between NNTs, patient outcomes and adverse events [23, 30, 47] Limited exploration of consequences for nurses [26, 30, 31, 44, 48, 49, 53], limited analysis of the consequences of NNTs, especially related to missed nursing care [54] Limited cost analysis [24, 31, 40]	Reframing NNTs within a broader discourse on professional practice [25, 48, 49, 52] Exploring how clear job descriptions and appropriate support staff allocation can reduce NNTs [23, 26, 27, 38, 44, 45, 51, 53] Evaluating technology‐based strategies to reduce administrative burden and enhance time for clinical care [31, 45, 50] Conducting multicentre, large‐scale studies to map types of NNTs and time spent on them [21, 53] Developing validated tools and instruments to capture the complexity and typologies of NNTs [38, 48] Implementing benchmarking strategies to identify time spent on non‐value‐added activities [29] Investigating NNTs to improve both cost efficiency and care quality [31]	Reconfigure staffing, skill mix and nurse–patient ratios to reduce the NNT burden [21, 26, 30, 39, 41, 42, 46–48] Redesign workflows, collaboration and support systems [27, 29–31, 48, 49, 51, 52] Enhancing professional nursing roles and practice [21, 25, 38, 39, 49, 52, 53] Providing legislative measures to regulate nurse workload [26]

*Note:* PAGER, Patterns, Advances, Gaps, Evidence for practice, Research recommendations.

Abbreviation: NNT, non‐nursing task.

#### 3.3.2. Research Recommendations

Future research should prioritise (Table 5) reframing NNTs within a broader discourse on professional practice [25, 48, 49, 52] and developing and validating tools to assess NNTs [38, 48]. Multi‐centre, large‐scale studies [21, 53] and benchmarking strategies [29] are also required, including assessing the role of support staff in task redistribution [23, 26, 27, 38, 45, 51, 53], how job role clarity can mitigate the occurrence of NNTs [23, 38, 44] and the impact of technology‐based solutions in healthcare for reducing administrative tasks, as reported in some studies [31, 45, 50].

#### 3.3.3. Policy Recommendations

To address the NNT issue, the studies (Table 5) recommend reconsidering the skill mix and staff composition [26, 30, 39, 41, 42, 47, 48] and the nurse–patient ratio [21, 26, 46], redesigning workflows [29, 30, 48, 49], strengthening interprofessional collaboration [48, 49, 51] and developing support systems [27, 31, 52]. One study recommended that policymakers establish legislative initiatives to regulate nurses’ workloads and reduce nurses’ NNT responsibilities [26]. Enhancing the nurses’ role and identity through education, managerial supervision and professional development has also been recommended to prevent or mitigate NNTs [21, 25, 38, 39, 49, 52, 53].

## 4. Discussion

### 4.1. Study Patterns

To the best of our knowledge, this is the first review to map four decades of research on NNTs across all healthcare settings, as the two available reviews have focused on studies of the nature of nursing work in long‐term care facilities [23] and the nature of non‐nursing duties in operating theatres [40]. This gap in the literature may be attributed to the conceptual complexity of the topic and the evolving nature of the nursing profession itself, which complicate efforts to synthesise and categorise what constitutes a nursing intervention and what does not.

Over the past 40 years, 28 studies on NNTs have been published, averaging one per year, with peaks in 2019 and 2021. Despite a certain degree of continuity in these investigations, the topic has not attracted extensive attention, with more studies conducted in Europe and Asia, while the earliest originated in the United Kingdom and the United States. Differences across countries in the evolution of the nursing role [55], the availability of auxiliary personnel and the level of organisational support may have influenced the need to investigate NNTs. In countries where the profession is still evolving from a predominantly auxiliary role to a more autonomous and professional one, or where nurses are expected to compensate for shortcomings in healthcare systems by undertaking activities beyond their professional remit (e.g. administrative tasks), investigating NNTs remains particularly relevant [56].

To date, studies have primarily focused on measuring the extent and frequency of NNTs and on nurses’ perceptions and the impact on their profession; less attention has been paid to identifying their underlying causes or evaluating interventions aimed at reducing their occurrence. As evidenced by the predominance of cross‐sectional designs, the scientific output in this field has been largely descriptive rather than intervention oriented. All studies involved registered nurses, mainly in hospital settings, although five also included nursing assistants or other support staff. Including professionals whose roles overlap with those of nurses when investigating NNTs may provide a more comprehensive understanding of task distribution and the factors influencing their occurrence.

Data collection has largely relied on paper‐based self‐report instruments, using a range of ad hoc tools, which limits comparability across studies and hinders the accumulation of robust evidence in this field.

### 4.2. Advances

#### 4.2.1. Concepts and Definitions

Over the last 4 decades, the terminology has evolved from ‘non‐nursing duties’ in earlier years to ‘NNTs’ more recently, alongside other minor alternatives. In early studies, ‘non‐nursing duties’ was often used to distinguish the core tasks of nursing from ancillary work; in the 1990s, ‘non‐RN nursing’ focused on the professional identity of nurses delineated tasks that did not require their level of qualification. Around the same time, influenced by lean management principles [57], terms such as ‘non‐value‐added tasks/time’ framed the issue in terms of efficiency and value rather than a strict delineation of professional roles. This linguistic evolution reflects the changing identity of the nursing profession [58], as well as a shift from functional classifications of tasks to value‐ and systems‐based perspectives.

Moreover, findings suggest three main reflections:

- Most terminology used to date revolves around observable, behaviourally defined activities and therefore focuses on concrete and visible tasks when distinguishing between appropriate and inappropriate use of nursing time. However, nursing work cannot be described solely as concrete actions or as a set of isolated tasks [59].
- In terms of conceptualisation, the definitions provided by studies share several commonalities: (a) they focus on tasks that are outside or below the scope of nursing practice; (b) they refer to activities that do not require nurses’ knowledge or qualifications; (c) they exclude activities involving direct patient care; and (d) they imply actions that could be delegated. However, the scope of nursing activities is neither static nor universally defined. It varies between countries, changes with professional development (e.g. from novice to expert; advanced nursing roles) and is influenced by evolving healthcare systems. For example, nursing assistants are increasingly taking on traditional nursing tasks, and other roles in the healthcare system are also being expanded and redefined [60]. This dynamic poses a challenge for both the conceptual and operational definition of NNTs. In addition, even criteria such as ‘not associated with direct care’ are becoming blurred, especially with the advent of digital care measures. The definition of NNTs or duties that could be delegated is also ambiguous, particularly when nurses remain responsible for the outcomes.
- Most of the terms used begin with the prefix ‘non’, defining the concept by what it is not, whether in relation to tasks, duties, roles (e.g. RN) or functions. In clinical nursing practice, however, the appropriateness of an intervention is determined not solely by the nature of the task but by the patient’s condition and the clinical intent behind the action. Activities may be nursing or non‐nursing and may become appropriate depending on patients’ needs, the scope of the activity and the context in which it is carried out. For example, answering a telephone call in a rigid, task‐oriented environment may be considered an NNT, whereas the same task in an oncology unit may represent the highest level of personalised care and case coordination. Moreover, tasks extend beyond traditional nursing boundaries are described as task shifting or task sharing [61], highlighting the need for greater clarity between NNT concepts, definitions and other related concepts.

As these reflections suggest, further efforts are needed to enhance conceptual clarity in the field, thereby improving our ability to understand and measure NNTs and to design strategies to mitigate them while avoiding conflation of the professional nature, value and delivery of nursing work when interpreting NNTs.

#### 4.2.2. Types, Time and Temporal Trends of NNTs

Despite technological advances and evolving nursing roles over the past 4 decades, studies have documented a relatively stable overall profile of NNTs. Clerical and administrative duties, highlighted in early studies, continue to receive attention, indicating that they remain unresolved, whereas housekeeping tasks have become less common, reflecting changes in nursing roles. More recently, studies [48, 53] have examined tasks normally expected of other healthcare professionals. Overall, NNTs investigated to date have concerned (1) tasks that do not require nursing knowledge or skills and (2) tasks typically performed by professionals with comparable levels of education (e.g. physiotherapists) [48, 53]. The first category mainly involves low‐skilled administrative or support activities that divert nurses from patient care, whereas the latter keeps nurses close to patients despite extending beyond their formal scope of practice. To the best of our knowledge, NNT‐related issues have not been documented in other healthcare professions, suggesting that nursing has historically been characterised by exceptional role flexibility.

Although earlier studies reported wide variability, more recent evidence consistently indicates that NNTs account for approximately 20%–30% of nurses’ working time, suggesting that most of their working time remains devoted to nursing activities [62]. Nevertheless, preventing NNTs could release substantial nursing time for direct patient care.

#### 4.2.3. Antecedents

Alongside the numerous studies investigating unit‐level antecedents, such as staffing, administrative and leadership shortages that compel nurses to fill these gaps, some studies have also explored antecedents at the interprofessional level, suggesting that this phenomenon reflects a complex interplay across multiple levels. Nurses have been shown to compensate for gaps in the work of other healthcare professionals, resulting in an informal redistribution of tasks. According to the International Council of Nurses (2025), nurses play a central role not only in providing direct patient care but also in supervising, coordinating and delegating care activities to others [63]. Because nursing work processes are closely integrated with those of other healthcare professionals, effective models of care delivery should promote interprofessional collaboration while ensuring that responsibilities are aligned with professional qualifications, competencies and workload priorities [64–66].

At the individual level, the limited available evidence suggests that nurses often experience difficulties in delegating activities, and that uncertainty regarding the nursing role, as well as the education they have received, influences this. At the same time, their strong professional commitment and the moral implications of leaving necessary care undone may lead them to undertake tasks beyond their professional scope, even when they recognise that these activities could be delegated [67]. As healthcare professionals who spend the greatest amount of time in direct contact with patients, nurses may feel an intensified sense of moral responsibility that prompts them to compensate for deficiencies wherever they arise. Furthermore, the recently introduced concept of entanglement in nursing care, which describes nursing activities as highly interdependent and difficult to separate without affecting the meaning and quality of care [68], may further explain why nurses engage in NNTs. When care activities are tightly interconnected, distinguishing between nursing and non‐nursing responsibilities becomes increasingly challenging in everyday clinical practice, making task compensation a natural consequence of efforts to maintain the continuity and safety of patient care.

#### 4.2.4. Consequences

Although NNTs may help ensure short‐term organisational flexibility and provide temporary relief during acute staffing shortages, systematically assigning them to nurses can have negative consequences for patients, nurses and healthcare organisations. The most frequently investigated impact concerns nurses’ well‐being, followed by patient outcomes and, less frequently, consequences for the healthcare system. These effects may also extend to the nursing profession more broadly, undermining its attractiveness, public image and professional integrity, particularly when patients’ expectations of nursing care are not met [69]. However, most of the included studies employed cross‐sectional designs, and data were often collected using self‐report questionnaires or other tools. Therefore, the documented consequences should not be interpreted as being directly caused by NNTs. Rather, these findings should be regarded as insights into the potential impact of such activities and as a basis for reflecting on the burden that NNTs may impose in terms of consequences for patients, nurses themselves and healthcare organisations. Research and targeted initiatives are therefore needed to identify effective strategies to mitigate this silent phenomenon and counter the tendency to rely on NNTs as a routine form of organisational flexibility or compensation [70].

### 4.3. Gaps, Research and Policy Recommendations

Overall, the identified gaps, research priorities and policy recommendations appear closely aligned, collectively providing a blueprint for action in the coming years. Progress should begin with a more consistent conceptualisation and definition of NNTs, with future research also considering recent insights into their entanglement with nurses’ professional roles, organisational processes, and contextual conditions. Building on this conceptual foundation, robust multicentre, large‐scale studies are needed not only to describe the prevalence, type and distribution of NNTs across settings and shifts but, more importantly, to determine how they can be effectively prevented or mitigated and to evaluate their consequences for patients, nurses, organisations and costs. This requires moving the field from predominantly descriptive research towards intervention and implementation studies capable of testing solutions in real‐world settings. At policy and organisational levels, priorities should similarly shift from accommodating NNTs as an implicit source of workforce flexibility to addressing the conditions that generate them, through appropriate staffing and skill mix, clearer task allocation and professional roles, workflow redesign, effective use of support staff and technology and stronger interprofessional collaboration. Taken together, these priorities suggest that future efforts should not merely document NNTs but develop, implement and evaluate strategies that protect nurses’ professional scope of practice while ensuring safe, sustainable and efficient models of care.

### 4.4. Implications for Nursing Management

Overall, findings have several implications for nursing management. First, nurse managers should consider organisational conditions that contribute to the occurrence of NNTs, including staffing shortages, workload, skill mix, limited availability of support staff and unclear job descriptions. Monitoring the distribution of nurses’ activities may identify recurrent patterns of NNTs and inform targeted organisational responses. Second, nurse managers should promote greater clarity in professional roles and responsibilities, particularly where tasks overlap across professional groups. Clear task allocation, appropriate delegation processes and interprofessional communication may help reduce unnecessary duplication and informal redistribution of work. Workflow redesign and the appropriate use of support staff and technology could reduce administrative and auxiliary activities. Finally, nursing management should adopt a contextual approach when addressing NNTs. Not all activities traditionally classified as non‐nursing are necessarily inappropriate, as their relevance depend on patients’ needs, nurses’ responsibilities and the process of care. Therefore, management strategies should focus on ensuring that nurses’ time is appropriately allocated to activities requiring nursing knowledge, clinical judgement and professional competence.

### 4.5. Limitations

This scoping review has several limitations. Firstly, although a comprehensive search strategy was applied across multiple databases, only peer‐reviewed literature written in English or Italian was included, thereby excluding potentially relevant grey literature and introducing possible language bias. Secondly, the heterogeneity of terminology and conceptual frameworks in the studies posed a challenge for constructing the search strings and retrieving studies, leading to an under‐representation of studies using alternative descriptors. In this regard, the search strategy employed may not have identified all studies examining NNTs, given the variety of alternative terms used in the field (e.g. work below scope of practice). Thirdly, as the review aims to map various patterns, evidence, antecedents and consequences, the heterogeneity of the studies and terminology may have affected the findings in both the narrative synthesis and the thematic analysis. Moreover, the concept of NNTs is inherently context dependent. There is considerable variation in NNTs between countries [56], and this is important to consider when generalising the results of the scoping review.

## 5. Conclusions

To the best of our knowledge, this is the first scoping review to map 40 years of research on NNTs, showing that they remain a persistent feature of nursing practice across healthcare settings and continue to consume a substantial proportion of nurses’ working time. Although undertaking NNTs may provide short‐term organisational flexibility, help compensate for staffing or service gaps and reflect issues in interprofessional collaboration or individual nurses’ attitudes and competence, their systematic use may have negative long‐term consequences for nurses, patients and healthcare organisations.

The knowledge mapped to date, together with the identified gaps and recommendations, provides a blueprint for future action. Documented priorities include establishing shared conceptual definitions and standardised measurement approaches and developing and evaluating interventional studies to assess the effectiveness of preventive or mitigating NNTs based on redesigning work processes, staffing models, skill mix and task allocation in line with professional competencies and patient needs. Organisational and policy strategies should ultimately protect nurses’ time for activities that require nursing knowledge, judgement and competence. Overall, addressing NNTs is about recognising the value of nursing work and ensuring that nurses are able to devote their time to high‐quality, patient‐centred and safe care. Consequently, action is needed at multiple, interconnected levels. Nurse educators should consider the occurrence of NNTs when selecting settings for clinical placements and identify clinical mentors who can foster a strong professional identity and a clear understanding of the nursing role. Researchers should incorporate the priorities identified in this review into future research agendas while nurse leaders should promote policies that protect nurses’ time for activities requiring their professional competencies. At the clinical practice level, systematic auditing should identify settings in which NNTs recur consistently and inform targeted action to address their underlying causes. Taken together, these efforts could generate a coordinated, system‐wide strategy in which education, research, management and clinical practice converge on a common goal: protecting nurses’ time and ensuring that their professional expertise is devoted primarily to patient care.

## Author Contributions

Conceptualization: E.B., A.D.M., A.P.; data curation: G.M., E.B., S.C., G.D., A.C., E.A., F.C., M.C., A.D.M., A.P.

Formal analysis: G.M., S.C., G.D., A.P. Funding acquisition/investigation: G.M., E.B., S.C., G.D., A.C., G.M., A.P. Methodology: G.M., E.B., S.C., G.D., A.C., E.A., F.C., M.C., A.D.M., A.P. Project administration: A.P.

Resources: G.M., A.C. Software/supervision: A.P. Validation: G.M., E.B., S.C., G.D., A.C., E.A., F.C., M.C., A.D.M., A.P. Visualization: G.M., A.P. Writing–original draft: G.M., E.B., S.C., G.D., A.P. Writing–review and editing: G.M., E.B., S.C., G.D., A.C., E.A., F.C., G.M., M.C., A.D.M., A.P.

## Funding

This study did not receive any grants from funding agencies like government, private or nonprofit organisations.

## Conflicts of Interest

The authors declare no conflicts of interest.

## Supporting Information

Additional supporting information can be found online in the Supporting Information section.

## Supporting information


**Supporting Information** Supporting Table 1. Members of RICI Group, Italy. Supporting Table 2. Preferred Reporting Items for Systematic reviews and Meta‐Analyses extension for Scoping Reviews Checklist. Supporting Table 3. Studies not retrieved in their full‐text. Supporting Table 4. Studies included: data extraction.

## Data Availability

Data sharing is not applicable to this article as no datasets were generated or analysed during the current study.
