# Supplementary material for: Patterns, Evidence, Gaps and Recommendations Across Studies of Non‐Nursing Tasks: Findings From a Scoping Review
Source: J Nurs Manag. 2026 Oct 23;2026:9885933. doi: 10.1155/jonm/9885933 (PMC13692704; doi:10.1155/jonm/9885933)
Supplement: Supplementary file 1 [file JONM-2026-9885933-s001.zip › Supplementary Table 1_R3.docx]

**Supplementary Table 1.** Members of RICI Group, Italy‡

**Universities, Departments and Members**

*University of Udine*, Department of Medicine, Udine, Italy

Alvisa Palese, Stefania Chiappinotto, Alessandro Galazzi, Federico Fonda, Sara Dentice, Chiara Moreal, Gaia Dussi, Gaia Magro, Chiara Visintini, Erica Visintini

*University of Torino*, Department of Clinical and Biological Sciences, Torino, Italy

Valerio Dimonte, Paola Di Giulio, Sara Campagna, Beatrice Albanesi, Marco Clari, Alessio Conti, Silvia Gonella, Daniela Berardinelli, Elena Casabona, Federica Fenoglio, Federica Riva Rovedda, Elena Viottini, Jacopo Olagnero

*University of Piemonte Orientale*, Department of Translational Medicine, Novara, Italy

Alberto Dal Molin, Erika Bassi, Ines Basso, Erica Busca, Isabella Santomauro

*University of Verona*, Department of Diagnostics and Public Health, Verona, Italy

Luisa Saiani, Federica Canzan, Elisa Ambrosi, Giulia Marini, Michela Flippi, Arianna Caliaro

Legend. RICI, Ricerca Infermieristica Clinica e per l’Innovazione organizzativa e formativa (Clinical Nursing Research and Organisational and Educational Innovation)

‡ At the time of the research project
