# Supplementary material for: Patterns, Evidence, Gaps and Recommendations Across Studies of Non‐Nursing Tasks: Findings From a Scoping Review
Source: J Nurs Manag. 2026 Oct 23;2026:9885933. doi: 10.1155/jonm/9885933 (PMC13692704; doi:10.1155/jonm/9885933)
Supplement: Supplementary file 1 [file JONM-2026-9885933-s001.zip › Supplementary Table 3_R3.docx]

**Supplementary Table 3**. Studies not retrieved in their full-text

1) J. Robbins. “On Saying ‘No’ to Non-Nursing Duties.” *Pennsylvania Nurse* 32, no. 0 (n.d.).
2) No author listed, “Non-Nursing Work Still Continues,” *RANF Review* 2, no. 12 (1970), https://pubmed.ncbi.nlm.nih.gov/5211757/.
3) P. S. Jordan. Time Spent Performing Non-Nursing Tasks and Job Satisfaction of Certified Nephrology Nurses. *Thesis, Texas Woman’s University*, (1991). [https://hdl.handle.net/11274/12980](https://hdl.handle.net/11274/12980?utm_source=chatgpt.com)
4) R. C. Oleksij, “What Do They Mean: Non Nursing Duties?,” *RN* 27 (1968): 37+, 64.
5) R. M. McNamara. “Nursing Burn-out Caused by Demands of Non-Nursing Tasks, Says Pres-Elect.” *One to One* 2, no. 0 (n.d.)
