# Supplementary material for: Patterns, Evidence, Gaps and Recommendations Across Studies of Non‐Nursing Tasks: Findings From a Scoping Review
Source: J Nurs Manag. 2026 Oct 23;2026:9885933. doi: 10.1155/jonm/9885933 (PMC13692704; doi:10.1155/jonm/9885933)
Supplement: Supplementary file 1 [file JONM-2026-9885933-s001.zip › Supplementary Table 4_R3.docx]

**Supplementary Table 4**. Studies included: data extraction (N=28)

|  |  |  |  | **Non-Nursing Tasks** | | | |  |
| --- | --- | --- | --- | --- | --- | --- | --- | --- |
| **Authors**  **Year**  **Country** | **Study aim(s)** | **Study design** | **Setting, participants, data collection tools and process** | **Conceptual definitions** | **Main features (e.g. frequency, type)** | **Antecedents** | **Consequences** | **Gaps/Recommendations** |
| Ahmadishad et al.  2019  Iran [21] | To measure care and noncare-related activities among critical care nurses and to determine their contributing factors | Cross-sectional observational time and motion study | Critical care units  70 nurses  Nurse and patient characteristics questionnaire and care and noncare-related activities checklist  Literature review using critical care nurses’ job description  Time and motion observation technique during morning shift for 2 months | **Noncare-related activities:** not related to patient care but significantly affect the nursing workload  **Unit-related tasks**: the management of the unit and pave the way for care provision to all patient  **Personal activities**: related to nurses’ personal lives and mostly include their activities of daily living | 110.9 min (30.8%) on noncare-related activities in morning shift  76.5% on personal activities  23.5% unit-related activities  Correlation to personal activities and units/patient-ratio  Correlation to unit-related activities with patient-ratio  Nurses who provide care just to one patient spent greater amount of their working time on unit-related tasks than other nurses | Type of unit Nurse-patient ratio  Level of their professional commitment and behavioural patterns  Weakness of managerial supervision  Limited familiarity with job description  Organizational policy and patients’ characteristics | NR | To highlight the necessity of closer managerial supervision of nurses’ activities, in-service educations for nurses about effective time management, and determination of the most optimum nurse-patient ratio and task division schedule for nurses  Needing multicentre studies to assess the time distribution of nurses’ activities and its contributing personal, professional, and organizational factors |
| Al-Akash et al.  2024  Jordan [38] | To fill the knowledge gap about this topic both nationally and internationally  To explore the experiences and perspectives  of Jordanian nurses regarding NNTs within the healthcare context  To identify and classify the  various types of NNTs that nurses encounter  in their daily practice, including administrative duties,  patient transport, and other tasks traditionally performed  by non-nursing personnel  To examine the  impact of NNTs on nurses’ roles, workload, job satisfaction,  and overall well-being in hospitals  To investigate the root causes of NNTs within the healthcare system and understand their far-reaching  consequences for nurses and patients  To propose  potential solutions and interventions to mitigate  the challenges posed by NNTs and improve the quality of  nursing care and nurses’ working conditions in  hospitals | Qualitative exploratory study | Three expansive hospitals  38 experienced nurses  In-depth focus group interviews  Open-ended questions  designed to prompt participants to reflect on their understanding of NNTs and their impact on their work  Questions encouraged participants to share  their personal experiences, (positive and negative),  related to NNTs. Questions that solicited suggestions for mitigating the  prevalence of NNTs and potentially streamlining workflows  within Jordanian healthcare settings | **NNTs:** can encompass a wide range of duties that fall outside the traditional scope of nursing practice | Type of NNT reported: clerk and management tasks, transporting tasks, and physician tasks  Administrative and clerk duties  Transporting patients to radiology  Obtaining medications from the pharmacy Significant time  time transporting blood, urine, and other samples  highlighting  challenges with the availability and responsiveness of  transporters.  Nurses assumed responsibilities typically attributed  to physiotherapists and respiratory therapists,  particularly in critical care departments. Nurses frequently took charge of inquiring about patients’  dietary preferences and checking for prescribed or special  diets in patient files | Clarity in defining nurses’ role  Staffing shortages and allocating sufficient resources  Workload  Role of management in task allocation  Transparency in task distribution  Confusion on nurses’ duties and responsibilities  Unclear communication channels  Role of university and bodies in building nurses’ personalities  Fair compensation when asked to perform  tasks outside their core responsibilities | Nurses’ effectiveness, productivity, and professional growth  Hindering ability to fully grasp clinical difficulties and make decision  Internal conflicts and demotivation  Long-term commitment to the nursing profession  Nurses and the quality of healthcare services | Participants also emphasized the importance of nurturing nurses’ professional identities during their academic training and mobilizing nursing unions to champion nurses’ rights  Through these proposed solutions, the theme advocates for comprehensive strategies aimed at streamlining task management, bolstering the nursing workforce, and empowering nurses to prioritize their core responsibilities  Rotation-based task assignment system  Augmenting the workforce by increasing the number of aid nurses, porters, and support personnel  Clear job descriptions and strict adherence to them as essential measures to reduce confusion and ambiguity surrounding NNTs  Transformative potential of creating clear job description and recognising the need for a fresh perspective on roles and responsibilities in addressing the complexities of NNTs  To better understand the contextual factors and consequences of NNTs experienced by nursing staff and students  To develop instruments measuring the complexity of NNTs is crucial for comprehensive assessment  Policy makers and decision-makers in the healthcare sector can leverage the findings of this research to guide the development of policies and the allocation of resources  Necessitating system-level changes to address underlying resource constraints  To fully understand the contextual factors and consequences of NNTs for nurses globally |
| Aldarawsheh et al.  2024  Jordan [26] | To investigate the perceptions of Jordanian nurses regarding NNTs and their consequences, as well as the underlying factors that contribute to occurrence of NNTs | A qualitative-exploratory approach | Three large hospitals  38 nurses in multiple groups based on shared characteristics  Focus group interview on which the discussion was focus on the idea of NNTs, the tasks performed considered the NNTs, NNTs impact on nurses’ performance and quality of care  Developed based on a literature review and consultation with experts | **NNTs:** do not require professional nursing training and should be assigned to other staff; they are considered a barrier to the delivery of nursing care management, as they are performed by professional nurses outside their scope of practice | More than 50% were NNTs’  Type of NNTs: management and clerical, transporting, physician tasks, tasks performed by other health professionals, and communicating and coordinating tasks | Staff shortage  Limited resources  Performed their NNTs as a favour of other colleagues  Weakness of nursing administration (leading to assignment of NNTs to nurses)  Weakness of education | Deterioration of nursing care Challenges to prioritize effectively or provide patient care  Impaired nursing care  Physical and mental health strain (physical exhaustion, feeling of stress, overwhelm, emotional distress)  Lack of efficiency, productivity,  professional development and the quality of patient care  Internal struggles and disheartenment among some nurses | To address staff shortages and ensure nurses have the necessary support to focus on their clinical duties  To optimize nurse-to-patient ratios and to investigate in staffing levels  To consider legislative measures to regulate nurse workload and limit non-nursing responsibilities  To incentivize institutions to prioritize direct patient care and address NNTs  To explore NNTs’ prevalence, develop interventions to reduce NNTs, and evaluate their impact on nurses and patient care  To train and educate nurses to effectively manage NNTs and advocate for their roles, skills and knowledge necessary to meet evolving healthcare demand |
| Bekker et al.  2015  South Africa [53] | To investigate the relationship between NNTs, nursing task left undone and job satisfaction among professional nurses in medical and surgical units in private and public hospitals | Cross-sectional survey | 60 medical and surgical units  1166 nurses  Sections of the questionnaire RN4CAST (section A, C and D) about job satisfaction, NNTs and nursing tasks left undone | **NNTs:** include all tasks performed by professional nurses that are below their scope of practice and are associated with tasks not related to direct patient care or tasks not requiring professional nursing skill during one nursing shift | Categories: delivering and retrieving food trays, housekeeping duties, transporting patients, ordering supplies, obtaining equipment, discharge referral and transport arrangements, routine phlebotomies, filling in for off-hours non-nursing service and clerical duties  Answering phones/clerical duties [mean (M)=1.81] arranging discharge referrals and transportation (M=1.38)  Performing non-nursing care (M=1.33)  Obtaining supplies and equipment (M=1.12)  Correlation between NNTs and nursing tasks left undone | High overall workload (High unit occupancy  Units with more than 25 beds (Nurse to patient ratio)  Unfavourable practice environment  Poorly functioning support services | Leave some nursing tasks left undone  Dissatisfied with their job when they performed some NNTs (cleaning patients’ room and equipment, filling in for non-nursing service not available off hours and obtaining supplies or equipment) | To consider what it is that professional nurses do and what they ought to do in terms of their skills, education level and expertise  Nurses feel NNTs as part of their workload  Nurse managers need to identify the tasks that professional nurses perform below their scope of practice to minimise NNTs and to ensure efficient support system |
| Fulbrook  1999  United Kingdom [39] | To provide a descriptive account of the activities that nursing  staff performed in the department  To quantify the amount of time spent on the activities performed  in a given period  To make proposals on the modification and development  of the Registered Sick Children’s Nurses (RSCN)’s role | Observational study | A paediatric outpatient department  Three RSCNs and one Health Care Support Worker (HCSW)  Non-participant observation and focused interviews  sample  3-hour sessions of non-participant observation of each staff member, followed by 1-hour focused interviews | **Non-nursing duties:** do not require nursing qualifications or clinical judgement and can be delegated to support staff. These include clerical duties, equipment maintenance, housekeeping, and basic measurements such as height and weight assessments | Non-nursing activities accounted for approximately 60% of staff time  Key categories included: housekeeping (5.4%), equipment maintenance (9.2%), equipment preparation (2.8%), measuring (13.5%), administrative tasks (9.5%), and incidental health promotion (0.6%). Such tasks were primarily executed by the HCSW but also occupied a significant proportion of the RSCNs' time | Understaffing of support roles  Lack of formal role delineation | Underutilisation of qualified nurses’ expertise  Reduced capacity for direct patient care  Potential compromise of care quality  Missed opportunities for nursing-led service development | To reallocate non-nursing duties to support personnel to maximise the efficiency and cost-effectiveness of registered nurses’ time  To develop and implement of nurse-led clinics for manageable paediatric conditions To introduce structured clinical supervision to enhance professional growth and reflective practice  To explore telephone advice services to support families and improve accessibility  To redesign paediatric outpatient departments’ services based on skill mix analysis and evidence-based innovations |
| Grosso, Tonet, Bernard, Corso, et al. 2019  Italy [40] | To explore the NNTs phenomenon, its antecedents and consequences according to the experience of nurses, to develop knowledge useful in highlighting its features and suggesting both professional and policy decisions | Descriptive qualitative study | Nursing Board of Belluno (Italy)  22 Nurses  Semi-structured interview  Available literature and  focus group with 11 members of Nursing Board with advance education and role of responsibilities developed the guide for conducting the interviews | **NNTs**: defined as tasks performed by clinical nurses below their scope of practice | Nurses can play out their role in two dimensions: outside and inside the role of other healthcare professions  Type: administrative activities that should be performed by secretaries or Nurse Aides (NAs); activities required less education (distribution lunch), the same amount (physiotherapist activities)  or higher (in-depth assessment of patient condition and communicate to the doctor, who decide the medication) education at university levels  Performing activities of other healthcare professional especially during weekends | 3 levels: Organizational (cost containment measures, shortcomings, skill mix revisions and unit routines and culture, implicitly force to perform some functions) Individual (desire to meet patients’ need and ensure their comfort and well-being, lack of communication and discussion)  Educational (vocational training to academic education) | 3 levels:  Patient and carer (Confusion and ambiguity, educating, surveying, monitoring and planning nursing care missed, patient and care detachment)  Professional (frustration, emotional stress and burnout of students, erosion of nursing competences, disaffection intention to leave)  Organizational (efficiency in compensating for the lack of resources, accumulation of extra hours due to the need to complete nursing care)  Feeling tension towards other healthcare workers who manifest less flexibility  Increased need of care | To invest in healthcare assistants has been limited over the years  Early forms of advances practice: inconsistencies in designing the workforce skill mix and shift patterns according to patients’ needs; expansion of nurses’ competences when they undertake diagnostic or treatment decision in the absence of physicians  To enhance nursing identity  To redesign the entire system  To educate all healthcare workers to cooperate, also organizational models should be redesigned to facilitate cooperative approaches  To redesign needs of organizational processes and settings  To provide interprofessional experiences, by also teaching moral obligation towards patients as a value to all healthcare workers  To rethinking the staff composition and their skill mix  To develop instrument to measure the complexity of the NNTs |
| Grosso, Tonet, Bernard, De Marchi, et al.  2019  Italy* [41] | To quantify the phenomenon of NNTs and describe the strategies implemented by nurses to prevent and/or limit their occurrence | Cross-sectional study | Nursing Board of Belluno (Italy)  743 Nurses  Questionnaire developed from prior qualitative phase  Questions included:  Percentage of shift spent on NNTs, strategies were used to avoid/limit them (yes/no), type and frequency of strategy use (Likert scale 1-4), Perceived effectiveness of each strategy (Likert scale 1-4)  Based on literature review, nurses’ letters to the Nursing Board, and focus groups | **NNTs:** defined as activities that should be performed by other staff members and do not require nursing knowledge or skills: e.g. retrieving meal trays, cleaning rooms, answering phones, secretarial work | 94.5% of nurses reported performing NNTs daily  Average time spent: 32.6% of the work shift  Consistent with previous studies estimating 35-62% of shift time  Only 40.5% reported using strategies  Specific actions:  Peer review of work organization: 60.7%  Overtime to compensate for lost nursing time: 59.7%  Group discussion of possible solutions: 59.6%  Refusing to perform NNTs: 59.3%  Documenting such tasks: 57.8%  Perceived Effectiveness:  Overtime: 2.21 (most effective)  Group discussion: 2.15  Reviewing organization: 2.20  Refusing NNTs: 1.75  Documenting tasks: 1.68 (least effective) | Lack of support and auxiliary staff  Changes in staff mix  Organizational structure and culture  Absence of non-nursing personnel in some work environments | Increased missed care  More time on indirect care  Decreased job satisfaction  Increased perceived time waste  Risk of burnout and team conflict | Only a minority of nurses take proactive steps to prevent NNTs  There may be a sense of resignation or belief that solutions are not within the nurses’ power  To explore strategies in different settings (macro, meso, micro levels)  To involve other professionals (e.g., doctors, support staff) in redesigning work processes  To promote nurses' professionalism rather than simply rejecting inappropriate tasks |
| Grosso et al.  2021  Italy* [42] | To describe the prevalence of the four different types of NNTs (auxiliary, administrative, allied health care professionals and medical)  To explore the reasons of auxiliary tasks as perceived by nurses | Cross-sectional study | Nursing Board of Belluno (Italy)  743 Nurses  Questionnaire survey about NNTs categories and frequency  Developed by 11 members of nursing board during five meeting, where a review of literature was performed | **NNTs**: to indicate activities not requiring nursing education that nurses must perform, as activities enacted by nurses “below their skill level”, as activities belonging to allied health care professionals  **Nonvalue-added (NVA) activities**: reported to consist in searching for and retrieving equipment, in escorting patients, doing paperwork and delivering supplies | Type: administrative (answer phone calls, scheduling meetings), auxiliary (room cleaning, delivering food, escorting patients)  Allied health care professional (mobilizing patients when physiotherapist is absent), medical  94.5% of nurses performed at least one type of NNTs  Most common:  Administrative (72.4%)  Auxiliary (66.7%)  Less common:  Allied health tasks (25.5%)  Medical tasks (18.6%)  Most frequent during morning shifts  Average time dedicated: 32.6% of total shift time  Tasks of allied professionals, while less frequent, took more time (35.2% of shift) | Working in hospitals (vs. community/residential) increases likelihood  Functional nursing model use  Perceived inadequacy of nursing resources  Overtime and excessive workload  Pressures from organizational culture  Perceived need to “compensate” for lack of resources [Odds Ratio (OR) 1.44]  Tasks decrease with unexpected clinical events (OR 0.58) or when adequate resources are present (OR 0.98) | Reduced job satisfaction  Increased intention to leave the unit  Professional burnout  Missed nursing care  Perception of wasted professional time | Few large-scale studies have explored all four types of NNTs simultaneously  Further research needed on antecedents and mechanisms behind NNTs  Strategies to improve nursing resource allocation  Organizational interventions to clarify nursing role boundaries  Education and policies to reduce role ambiguity  Promote task redistribution according to competencies  Starting discussion about the concept of NNTs |
| Hammad et al.  2021  Egypt [54] | To identify level and types of Missed Nursing Care  and to determine factors contributing to missed nursing  care, so that appropriate interventions can be developed  to reduce care omissions and thus improve quality and  safety of care | Cross-sectional design | 1762 beds teaching hospital  1211 Nurses  Schedule consisted of two tools: MISSCARE survey and the N4CAST survey  Tool to collected data and level of NNT with 9 task rating to a scale 3-point scale of never sometimes and often and frequency  Translation in Arabic by research team, assessed for face validity by panel of experts  Piloted on 20 nurses | **NNTs**: all tasks that are not related to direct care or tasks not requiring professional nursing skills | Type NNTs: delivering food trays, performing non-nursing care, arranging discharge referrals and transportation, phlebotomy, transporting patients within the hospital, cleaning patients’ rooms, filling-in for non-nursing services, obtaining supplies and performing clerical duties  Only cleaning patients’ rooms and equipment and filling in for non-nursing services and available on off hours related with Missed Nursing Care (MNC) | NR | MNC, but  Statistically NNTs showed negative weak correlation with the overall MNC | This unexpected finding  could be explained by the fact that nurses may  consider all the other NNTs to be part of their workload  Studies pointed out that nurses accept performing non-nursing duties, because these roles are founded on organizational as well as individual patient relationships |
| Hamm-Vida  1990  United States [49] | To examine the amount of time spent by the resource person on non-nursing activities, the impact of this time on the nursing division budget, and the ultimate cost to the hospital of the resource persons performing such duties | NR | Riverview Medical Centre 500-bed community hospital  Nurses (namely resource persons)  Tracking form completed daily | NR | Type: housekeeping, transportation, pharmacy, secretarial, medical records, electrocardiogram    From 1 to 5 for 7.5 hours shift on non-nursing activities  67.5 hours spent performing non-nursing activities during 11-7 shift (secretarial 92.52%)  35.82 hours during 3-11 shift (secretarial 54.76%)  3.84 hours spent performing non-nursing activities during 7-3 shift | NR | $113765 payment for the nursing division performed other department activities  $25740.84 annual cost an overall for performing non-nursing activities belonging to other departments | NR |
| Harrison & Nixon  2002  United Kingdom [43] | To identify, classify and categorize the activities undertaken  by nurses working in a seven-bed general  intensive care unit  To quantify the time spent on each category of activity  To identify the amount of time spent on non-nursing  classified activities  To compare the amount of time spent in non-nursing  activity with the current health care support worker  provision | Cross-sectional study | General Intensive Care Unit, 450 patients per years  RN  Self-reporting diary log sheets with pre-coded activity categories (direct nursing care, clerical nursing duties, patient assessment, non-nursing duties, time out)  Consultation of expert RN to review the tool for its development | NR | Nurses spent 4% of their total time on non-nursing duties  Morning weeks shift 3%  Late shift 6%  Cleaning bed spaces 5%  Night shift 3%  Type: cleaning and preparing bed spaces, ordering/putting away stores, non-nursing clerical duties, e.g. filling out requisition slips, telephone enquires not concerned with patients, checking general unit-based equipment/trolleys | Inadequate support staff coverage, particularly during late shifts | Reduced focus on direct patient care, inefficiency, and increased workload for RN | To expand the role of healthcare assistants to reduce non-nursing burdens on registered nurses  To conduct follow-up studies to measure the impact of hiring additional support staff on nursing efficiency and care quality  To investigate the use of technology to streamline documentation and administrative tasks |
| Hayes  1994  United States [25] | To define non-nursing functions, measure the extent RNs perform and their attitude toward them, and determine if changes in the performance of and attitudes about non-nursing functions could be accomplished among RNs | Quasi-experimental | General medical and surgical units  2 years  118 RNs  (83 at midpoint  62 at the end)  The Perfoming Non-Nursing Functions (NNFs) Scale (six items)  The Attitude About Performing Non-Nursing Functions Scale (other six items)  Both Likert-type scale  Test-retest reliability 20 RNs of general medical and surgical nursing units  Experimental group: revision of job description, support by head-nurses to identify NNFs, maintaining of responsibilities to the delegated to NAs | **NNFs**: non-clinical activities often performed by RNs that should or could be performed by support service staff or NAs | Type: clerical/receptionist, trips/errands off unit, housekeeping, making beds, dietary and pharmacy  At the beginning all reported usually or almost always  Experimental group: RNs disagreed more strongly at the end of the study that they should performed non-nursing functions that at the beginning (87% strongly disagreed or disagreed)  Agree more strongly at the end of the study that they should not perform NNFs  Performing and attitude about performing NNFs correlation with overall job satisfaction | Younger RNs  Shorter length of employment  Time  Lack in the role of NAs  Inexperience | Overall job satisfaction  Frustration  Clear professionals identify (experimental group) | To achieve a more highly professional practice, one in which NNFs are not imposed on RNs, but if done by RNs, completed for reasons in the best interest of patients |
| Hinno et al.  2011  Finland [44] | To investigate the relationships between nursing activities, nurse staffing, and adverse patient outcomes in hospital settings as perceived by registered nurses and compare the results between Finland and the Netherlands | Cross-sectional, descriptive questionnaire survey | Hospitals in Finland and the Netherlands, including academic-teaching and district hospitals  535 Finnish RN (response rate 44.9%)  334 Dutch RN (response rate 33.4%)  Questionnaire survey including items on nursing activities, staffing levels, and adverse patient outcomes  Subscales for nursing, non-nursing, and administrative activities  Tool developed by expert group, exploratory analysis for nursing activities  with validated language translations | NR | Type: delivering food, removing food ware, transporting patients, making beds, changing bed linen, administrative (making appointments for patients, ordering bedlinen and laboratory tests, making rosters and organising patients discharge papers)  Finnish nurses reported dissatisfaction with the availability of support services more often | Higher patient-to-nurse ratios, inadequate support services  Local context: nurses who work in a district hospitals had to carry out administrative tasks than  their colleagues in academic-teaching hospitals | Reduced time for crucial nursing activities | To develop strategies to improve nurse staffing patterns  To focus on unit-level context to analyse the effect of organization on nursing activities  To establish systematic adverse event reporting systems to reduce recall bias in studies |
| Hobgood et al.  2005  United States [28] | To describe how Emergency Department (ED) RNs allocate their time between tasks and identify how that task distribution changes with increases workload | Prospective direct observation time study | 60000-patient-per-years tertiary-care academic ED  RN staff (one nurse per observation)  2000, 2001, 2002  Nursing activities into 1 of 4 predefined tasks categories  7 non-consecutive 24 hour-period  Categories developed by 3 methods: literature search, unstructured interviews and unpublished pilot tasks classification study | **Non-RN care**: the tasks performed at or away from bedside that could be performed by ED personnel other than a licensed, credentialed RN or a licences physician | Type (non-RN care): computer order entry, training NA, receiving formal training, laboratory draws, vital sign check, respiratory therapy, obtaining ECGs, patient transport, room/bed cleaning, patient hygiene, preparing patient meals  Type (personal time): meal breaks, reading, non-patient relate conversation, restroom break  Time spent:  non-RN care: 6.8% Standard Deviation (SD)  personal time: 13.6 (19.1% SD 25.2 | Patient per nurse  ED acuity index  Nursing workload | NR | In future studies focusing on satisfaction, including also nursing specific demographics data would allow measures of experience, sex, and age to be correlated with time per task allocation satisfaction scores |
| Johnson  1989  United Kingdom [45] | To consolidate knowledge base on non-nursing duties  To identify the research previously carried out in the field  To select the most appropriate research methodology | Comprehensive literature review | Operational theatres  Nurses  Questionnaire, surveys and interviews  NR | **Non-nursing duties**: tasks not linked to direct patient care by the nurse, or which are another persons’ job, but which relieve the nurse, enabling her to the vote attention to direct patient care (MacLeod, 1985)  **Non nursing duties**: tasks that could otherwise have been performed by non-nursing personnel  **Characteristic**: the closer a task comes to directly affecting the patient, the more likely nurses believe it to be a nursing function (Davis, 1982) | Work of messengers, work of clerical staff, work of domestic staff (MacKay, 1988)  Laundry assistant, domestic, porter, catering assistant, clerk (MacLeod, 1985)  Housekeeping duties (Pilgrim 1988)  Housekeeping. clerical work, transportation, dietary, miscellaneous (Byrnes, 1982)  Restocking, cleaning and tiding, transporting, clerical duties and reordering (Davis 1982) | High patient turnover (MacLeod, 1985) | Loss of £130000 per annum (MacLeod, 1985)  Reduce job satisfaction (Davis, 1982) | More detailed research and appraisal of ancillary (MacLeod, 1985) |
| Johnson  1991  United Kingdom [46] | To investigate the proportion of time spent in the NNTs  To investigate theatre nurse practice in terms of time spent on the functions and tasks identified by discussion groups  To investigate the proportion of time theatre nurse, spend in the performance of NNTs as defined by discussion groups | Observational study | Hospital theatre  99 RNs working in theatre  List of tasks and categories  22 sessions of observations were made set at three-minute intervals with 96 hours of operating time (total of 7,804 observations)  Discussion group | NR | Type: restocking supplies of theatre wear, putting away stock deliveries, cleaning the operating room and equipment at the end of each session, answering telephones and bleeps, fetching and carrying non-urgent equipment, routine preparation of operating room furniture prior to commencement of session  11% of nurses’ time on NNTs  Increase in NNTs workload during evening and weekend shift | Lack of ancillary staff during evening and weekend shifts  Shortage of staff | NR | To improve the skill mix for the reduction of qualified nurse non-nursing workload  To place high priority on the circulating nurse role of supporting and controlling the scrubbed team |
| Kearney et al.  2016  Canada [51] | To assess and address the performance of non-nursing duties by nursing staff in a large regional health authority using a newly created tracking tool | Quality improvement initiative using a newly developed "Non-Nursing Duties Tracking Tool" and a survey of nursing managers | Hospitals, long-term care, and community care facilities  Frontline nursing staff, including RNs, licensed practical nurse(s) (LPNs), and patient care attendants  Nursing managers  "Non-Nursing Duties Tracking Tool" for documenting NNTs and a survey of nursing managers  The tracking tool recorded tasks across categories during a one-week period. Managers compiled data into master summary sheets and participated in a survey assessing the tool's effectiveness and resulting changes | **Non-nursing duties:** Tasks that do not require nursing skills, including clerical work, housekeeping, non- patient-related paper  work and transporting patients | Type: clerical, housekeeping, food services, clinical support, and transportation  Common tasks included answering phones, filing, cleaning equipment, preparing charts, and patient transport  These tasks accounted for significant time that could be reallocated to patient care | Limited availability of support staff (e.g., clerks, transporters, housekeepers) | Reduced time for direct patient care, increased potential for medical errors, and lower job satisfaction among nurses | Increase the number and roles of support staff, such as ward clerks and transporters, housekeeping, and delivering food to reduce nurses' non-nursing workload  Standardize training for support staff to enable them to take on additional responsibilities  Support staff should  be distributed more equitably throughout facilities  Enhance communication and collaboration between departments to improve task allocation and reduce overlap |
| Kiekkas et al.  2005  Greece [47] | To describe, categorize, and quantify nursing activities in the Postanesthesia Care Unit (PACU)  To determine if there are any differences in the use of using time among shifts  To determine if different nurse-patient rations and PACU experience of RN affect the use of time | Observational, work sampling study | PACU of the General University Hospital of Patras, Greece  RN, observed across all three shifts over a month  Form of categories and subcategories of nursing activities  Categories and subcategories were structured based on a review of literature, experience of the researchers and description of a typical day’s workflow  Descriptions were obtained through semi structured interviews  Nurses were consulted to check for appropriateness, completeness and clarity | **Non-nursing duties:** Tasks like cleaning spaces or transferring patients or equipment  Personal activities: attending training, breaks, personal use of telephone or staying in PACU without performing any duties | Non-nursing duties constituted 2.3% of nurses’ time (8.6% of time on clerical nursing duties and 9.3% on documentation)  Type: cleaning spaces or transferring patients or equipment | Inadequate nurse-patient ratios and staff shortages | Reduced time for patient assessment, direct care, and psychological support impacting care quality | To investigate strategies to reduce non-nursing duties for nurses, such as hiring support staff for clerical and administrative tasks  To improve nurse-patient ratios to allocate more time to direct care and patient assessment  To evaluate the effectiveness of implementing technological solutions like clinical information systems |
| Liu et al.  2021  China [27] | To compare changes on the quality of care, nurse job outcome, nursing work environment, non-professional tasks, and nursing care left undone in acute hospital in Guangzhou  To determinate the association of changes in the nursing work environment, in non-professional tasks, and nursing care left undone with nurse job outcome and quality of care with panel analysis | Perspective due stage panel analysis | 111 adult medical and surgical units from 23 hospital in 2014 and 108 units in 2018  1498 nurses in 2014  1424 nurses in 2018  1323 patients in 2014  1193 patients in 2018  7 item scale asking if responder had performed each task and how much time have they spend doing them on their last shift (4 points Likert-scale)  NR | **Non-professional tasks:** activities not requiring professional skills and not related to direct patient care | Nurses spent time on non-professional activities:  30.82% in 2014  27.27% in 2018  Type: discharge referral, coordination, non-professional service, cleanliness, equipment obtaining, patient transportation, food delivery | NR | NR | Nurses consider non-professional tasks to be part of their routine work due to lack of effective support system  To invest in support system establishment  To optimize efficient support services for improve the use of nurses’ time and energy  To improve nursing work environment and supporting nurses to engage in professional and direct patient care to improve nurse job outcomes and promote quality of care |
| MacLeod  1985  United Kingdom [24] | To identify how much time was being spent on non-nursing duties, the cost implications, possible workable solutions and to increase awareness of the problems | NR | Acute assessment/rehabilitation ward and a continuing care 15 and 18 beds respectively  Nurses  List of NNTs, total number of time spent on NNT and additional spaces were left to add extra task  Developing the list after discussion with all nurses involved  The essential information was transposed into graphic form | **Non-nursing duties**: task (or tasks) not linked to direct patient care by the nurse, or which is another person’s job, but which relieves the nurse, enabling her to devote attention to direct patient care | Type: patients clothing washing, folding, fetching/carrying from stores, packing up/moving rubbish/laundry bags, clearing tables after meals, unpacking laundry, dusting/cleaning bed/lockers, sorting and filing notes, taking patients to rehabilitation, catering chores (buttering bread, baking eggs) general errands  0.74 and 0.69 Whole Time Equivalent is lost to patient care  Marked variation between two to seven hours a day, which increase in the weekend  £130000 per annum represents the amount of the nursing budget spent on paying nursing to perform the work as ancillaries | Nursing shortage  Workload  Patient turnover  Lack of ancillary support  Pace of work  Level of education to performing non-nursing duties | Quality of care  Patient care  Increasing of cost of healthcare professionals | To examine by research team of the whole area of non-nursing duties |
| McCloskey  2015  Canada [29] | To determine how care providers spend their time in nursing homes and to identify if variations exist across homes, shifts and licensure level. Understanding how care providers spend their time may help identify opportunities to improve efficiencies within the nursing home setting | Multi-centre cross-sectional observational workflow study | Seven different nursing homes  RN, LPN, resident aides  June-August 2012  July-August 2013  Observation a range of activities  Care activities were identified and divided into the categories (direct care, indirect care, non-value added and other)  Established operational definitions of each activity and a pilot run to ensure all possible activities | **Non-value-added activities**: included those activities that did not impact care, such as waiting or looking for equipment | Type: look for chart, get/drop linen look for caregiver, restocking, walking, waiting, get/drop equip  RN spent between 3.5% and 28.9% (mean 13.9%) in non-value-added activities (67.7% walking around the unit and 22.0% looking for charts) (restocking, searching for equipment and supplies, distributing linen, and looking for other care providers)  LPN spent 18.5% (mean) in non-value activities; 59.5% of this time involved walking around the unit, 13.5% looking for another healthcare provider, 9.5% retrieving linen | NR | NR | To examine the work process in the homes with high time in non-value added, compared with homes with low time in non-value-added activities (benchmarking) |
| Montayre & Montayre  2017  Authors' affiliation: New Zealand Studies reviewed: United States  Canada Australia United Kingdom Belgium Switzerland [23] | To present a contemporary perspective of RNs’ work in long-term care (LTC) facilities, focusing on specific tasks and defining the characteristics of RNs' roles | Integrative review | LTC facilities across multiple countries  Studies reviewed involved various nursing roles, including RNs, licensed practical nurses, care assistants, and other ancillary staff  Work sampling, observational methods, self-report forms, questionnaires, and semi-structured interviews.  Tasks and activities performed by RNs in LTC facilities, categorized into direct care, indirect care, non-nursing activities and expanded and overlapping roles | **Non-nursing activities**: Tasks beyond traditional RN roles, including managing inventories, scheduling, and conducting evaluations | NNTs comprised activities expected of other staff (e.g., healthcare assistants, clerks, administrative staff), such as restocking supplies, finding equipment, doing inventories and managing resources  RNs often took on expanded roles due to unclear work boundaries, performing tasks outside their clinical scope | Poor role clarity, inadequate delegation, and staffing shortages in LTC settings | Overlapping roles, reduced focus on direct care, and erosion of RNs’ professional expertise | To clarify work roles between RNs and other LTC staff to improve efficiency and care quality  To investigate the impact of delegating RN-specific tasks to less qualified staff on safety and care standards  To promote career opportunities in LTC nursing through curriculum integration of specific RN LTC roles  To examine the effects of task interchangeability on RNs’ professional roles and patient outcomes |
| Norrie  1997  United Kingdom [48] | To investigate, quantify and categorise the workload of intensive care nurses involved in patient care in a United Kingdom Intensive Care Unit (ICU)  To pilot a simple tool that can be used for comparison with previous studies | Cross-sectional study | 12-bedded adult cardiothoracic ICU  36 RNs  Questionnaire with 5 categories of activities and their content  10 members staff involved in the development of research tool giving feedback and comments | **Non-nursing duties:** work that did not require the skills of a trained nurse to be completed (e.g. cleaning and preparing bed spaces, ordering stores, sundry clerical duties and answering telephones) | Type: cleaning and re-provisioning of bed spaces, the replacement of single use items such as oxygen therapy, fluid administration giving sets and ventilator tubing and the refurbishment of prescription and observation charts.  Total of 82% of nurses’ time undertaking appropriate nurse duties, in comparison with only 7% being used in non-nursing duties  Range of non-nursing duties from 3% to 14% | High turnover of patients  Lack of support workers in morning shift | NR | To explore what grades of staff would be most appropriate to carry out specific activities  To explore if the skill mix of staff employed is appropriate for the type of activities being carried out  Support workers could be further used to minimise their non-nursing duties, which may prove to be cost-effective |
| Sabo  1990  Canada [52] | To determine tasks the nurse was presently performing that could be done by other personnel perspective | NR | Hospital for Sick children  148 nurses  Questionnaires (with open-ended final question) and interview tool  Existing knowledge of staff to identify actual and potential non-nursing activities  Activities were grouped and became a framework of questionnaire  Under each category staff were asked to list any other activity they perform that were not identify | NR | Type: environmental, clerical, transportation, administrate, patient-related and other professional services  Number of clerical tasks reported: 13244 for answering phones, completing requisitions, taking messages  Number of patient-related tasks reported: 7472 for delivering and collecting of dietary trays and pursuing residents to write orders  Same categories increased in weekend shifts  Findings from open ended: non-professional services unit nursing aides and unit clerk coverage  Interview data provide similar information to the questionnaire findings | Shortage of nurses | Dissatisfaction with nurses’ work environment  Burnout  Turnover  No responses to patients’ needs | Each unit required their own aide, who support the nurses in stocking and maintain unit supplies, cleaning unit equipment, some transportation duties and general maintenance of unit environment  To develop the job description of unit clerk  To review the role of volunteers and the environmental service (e.g. physiotherapy, pharmacy) to provide and meet the patient and unit needs |
| Storfjell et al.  2009  United States [31] | To determine the amount and cost of NVA time in acute care nursing units, identify drivers of high-cost inefficiencies, and assess how these vary by unit type to inform care delivery model improvements | Cross-sectional study | Three hospitals (14 medical-surgical units  2 intensive care units, 3 labour & delivery units)  RNs, nursing assistants, unit clerks, clinical nurse specialists  Charge nurses  Activity analysis using the Easley-Storfjell Caseload/Workload Classification System  Activity-based costing (ABC) model applied to time allocations  Focus groups and interviews  3-6 participants per focus group from each discipline/unit | **NVA:** time spent on tasks that do not directly contribute to patient care or outcomes, includes delays, rework, unnecessary searching, and inefficient processes | 38% of nursing time in medical-surgical units is NVA  NVA costs per medical surgical unit: over $1 million/year  Top NVA cost categories: coordinating care, managing clinical records, documentation and equipment search  Patient care activities received less time: teaching and psychosocial support had lowest time allocation  RNs account for the majority of NVA wage costs (~$660k–$756k/year per unit)  Documentation and coordination were high-cost NVA categories | Fragmented workflows and frequent handoffs  Poor access to equipment, supplies, or medications  Difficult process of admission/transfer/discharge  Inefficient electronic health records (multiple systems, redundancy)  Inadequate interdisciplinary communication  Fluctuating staffing and time-consuming shift reports | Reduced time for direct patient care  Increased operational costs  Staff dissatisfaction and burnout  Increased risk of adverse events and lower patient safety  Documentation overload and repeated tasks  Disconnection between staffing and care needs | To redesign support systems and streamline workflows  To integrate IT systems to reduce duplication in documentation  Invest in activity-based management to understand workload and eliminate inefficiencies  Shift focus from increasing staff to improving system efficiency  Prioritize teaching and psychosocial care, currently undervalued  Aligning finance and nursing through accurate time-driven costing  Reducing NVA time is essential to improving both cost efficiency and care quality |
| Upenieks et al.  2007  United States [30] | To examine how RNs allocate time to various workload activities among RNs with prescribed staffing ratio and to evaluate the implications of California’s ratio on nursing units | A workflow methodology, prospective comparison design | One academic medical centre  Included: 2 telemetry units and 1 medical-surgical unit  11 RNs  Palm Pilot workflow tracker signalling every 10-15 minutes  Random selected RNs were followed by a researcher to record workflow activities  Categories:  Value-added activities (direct patient benefit)  Necessary activities (e.g., documentation, care planning)  NVA activities (e.g., waiting, searching for equipment)  Each activity was also classified by location and nature (direct care, indirect care, documentation, waste, etc.) | **NVA tasks:** Activities that do not directly benefit the patient and are not necessary for care delivery (e.g., waiting, delays, looking for equipment or staff) | Non-value-added time ranged from 16.7% to 21% of nurses' shift across units  Nurses spent only 25-32% of their time on direct care activities  Waste and inefficiencies (e.g., hunting for equipment, waiting) constituted 3.5-5%  Documentation time: 16-24% of total work time  Workload patterns varied significantly across similar units, suggesting staffing ratios alone don’t reflect workload | Inadequate inclusion of support staff in staffing calculations  Physical layout of units (e.g., distance to nurses' station)  Lack of standardization in patient classification systems  Regulatory focus on ratios without accounting for task complexity or indirect care | Overburdened RNs with indirect/non-nursing duties  Reduced time for direct patient care  Inconsistency in workload distribution and care delivery quality  Risk of burnout and inefficiency due to high administrative/documentation burden | Staffing ratios should include ancillary roles to reflect real workload  Focus should shift from just ratios to "value-added care" that impacts patient outcomes  To implement workflow optimization to reduce non-value-added time  To recognize nursing as a dynamic, collaborative process rather than a fixed set of tasks  Need for unit-specific assessments due to variability in workflow, layout, and patient acuity |
| Yauger  1984  United States [22] | To describe and quantify the time, cost, and effect on patient care of nurses performing clerical non-nursing functions and the effects such activities had on patient care | Systematic survey | Medical Centre in America  Facility of 1300 beds, provide acute, intermediate, extended and domiciliary care  RN and LPN  Quality Assurance study  Form on which nurses were asked to list clerical non-nursing functions, time spent, patient care activities delayed, and patient-care activities not done | **Non-nursing clerical functions**: receptionist duties (answering questions and directing unit traffic) receiving and making telephone calls, and transcribing orders; all at a level not requiring nursing expertise | 657.4 hours in clerical non-nursing functions  3.5% of all certified nursing time  Range from 1.4% to 12% depending on the unit Largest % in acute care unit (80%)  Telephone duties (46%)  Transcribing order (35%)  Reception duties (19%) | Late afternoon and early evening (shift time) | Compromising patient care activities, both direct and indirect | To encourage nurses to refuse to do non-nursing functions, especially when performing such activities would interfere with patient care  Discrepancies between actual time spent in non-nursing function and the perception of nurses  Investigation about if nurses are using non-nursing clerical functions as an escape from the pressure of patient care  To understand who is taking care of patients when nurses are performing non-nursing functions |
| Yen et al.  2018  United States [50] | To quantify nurses' time allocation on communication, hands-on tasks, and locations; compare these allocations across different time blocks; identify multitasking and location traits; and examine phone call interruptions during a nursing shift | Time motion study | Medical-surgical unit at a Mid-west academic medical centre  15 RNs  TimeCaT, a validated electronic time capture tool  The observable nursing activities list was refined iteratively and finalized during the training and trial observation  11 type of communication, 32 hands-on tasks, and 14 locations  Observations were conducted during a 12-hour day shift, split into 4-hour blocks  NR | NR  Multitasking was defined as the observable performance of two or more tasks simultaneously, such as talking to a patient while preparing medication | Nurses spent approximately 10% of their time on delegable and non-nursing activities, such as transferring records or copying documents  Type of NNTs: food tray and housekeeping | Insufficient staffing, inattention by other departments or staff | Reduced time for patient care, nurse dissatisfaction, inefficiency | To improve nurses’ time allocation for high-quality care  To maximise the potential of the nurses’ workforce by supporting nurses to truly practice at the top of their license  To develop strategies for reducing NNTs and interruptions  Technological solutions to minimize phone interruptions |

Legend: ABC, activity-based costing; ED, emergency department; HCSW, health care support worker; ICU, intensive care unit; LPN, licensed practical nurse; LTC, long-term care; M, mean; MNC, missed nursing care; MISSCARE, missed nursing care; n, number; NA, nurses’ aide; NNF, non-nursing function; NNT, non-nursing task; NR, not reported; NVA, non-value-added; OR, odds ratio; PACU, postanesthesia care unit; RN, registered nurse; RN4CAST, Registered Nurse Forecasting; RSCN, registered sick children’s nurse; SD, standard deviation;

* Two studies of Grosso et al. [41,42], had the same sample of participants, but different study aims.
